# Supplementary material for: Mechanistic basis of antimicrobial resistance mediated by the phosphoethanolamine transferase MCR-1
Source: Nat Commun. 2025 Nov 26;16:10516. doi: 10.1038/s41467-025-65515-3 (PMC12658134; doi:10.1038/s41467-025-65515-3)
Supplement: Supplementary file 1 — Supplementary Information [file 41467_2025_65515_MOESM1_ESM.pdf]

Mechanistic basis of antimicrobial resistance mediated by the phosphoethanolamine transferase MCR-1

Allen P. Zinkle[1#], Mariana Bunoro-Batista[2#], Carmen M. Herrera[3], Satchal K. Erramilli[4], Brian Kloss[1], Khuram U. Ashraf[1], Kamil Nosol[4], Guozhi Zhang[5], Rosemary J. Cater[1,6], Michael T. Marty[5], Anthony A. Kossiakoff[4], M. Stephen Trent[3,*], Rie Nygaard[1,7,*], Phillip J. Stansfeld[2,*] & Filippo Mancia[1,*]

[1]Department of Physiology and Cellular Biophysics, Columbia University Irving Medical Center, New York, NY 10032, USA.

[2]School of Life Sciences and Department of Chemistry, University of Warwick, Coventry, UK.

[3]Department of Infectious Diseases, College of Veterinary Medicine, University of Georgia, Athens, GA 30602, USA.

[4]Department of Biochemistry and Molecular Biology, University of Chicago, Chicago, IL 60637, USA.

[5]Department of Chemistry and Biochemistry, University of Arizona, Tucson, AZ 85721, USA.

[6]Institute for Molecular Bioscience, University of Queensland, Brisbane, QLD, Australia.

[7]Department of Radiation Oncology, Weill Cornell Medical College, New York, NY 10065, USA.

[#]Denotes equal contribution

[*]Correspondence to be addressed to: strent@uga.edu (S.T.), rin7007@med.cornell.edu (R.N.), Phillip.Stansfeld@warwick.ac.uk (P.S.), fm123@cumc.columbia.edu (F.M.).

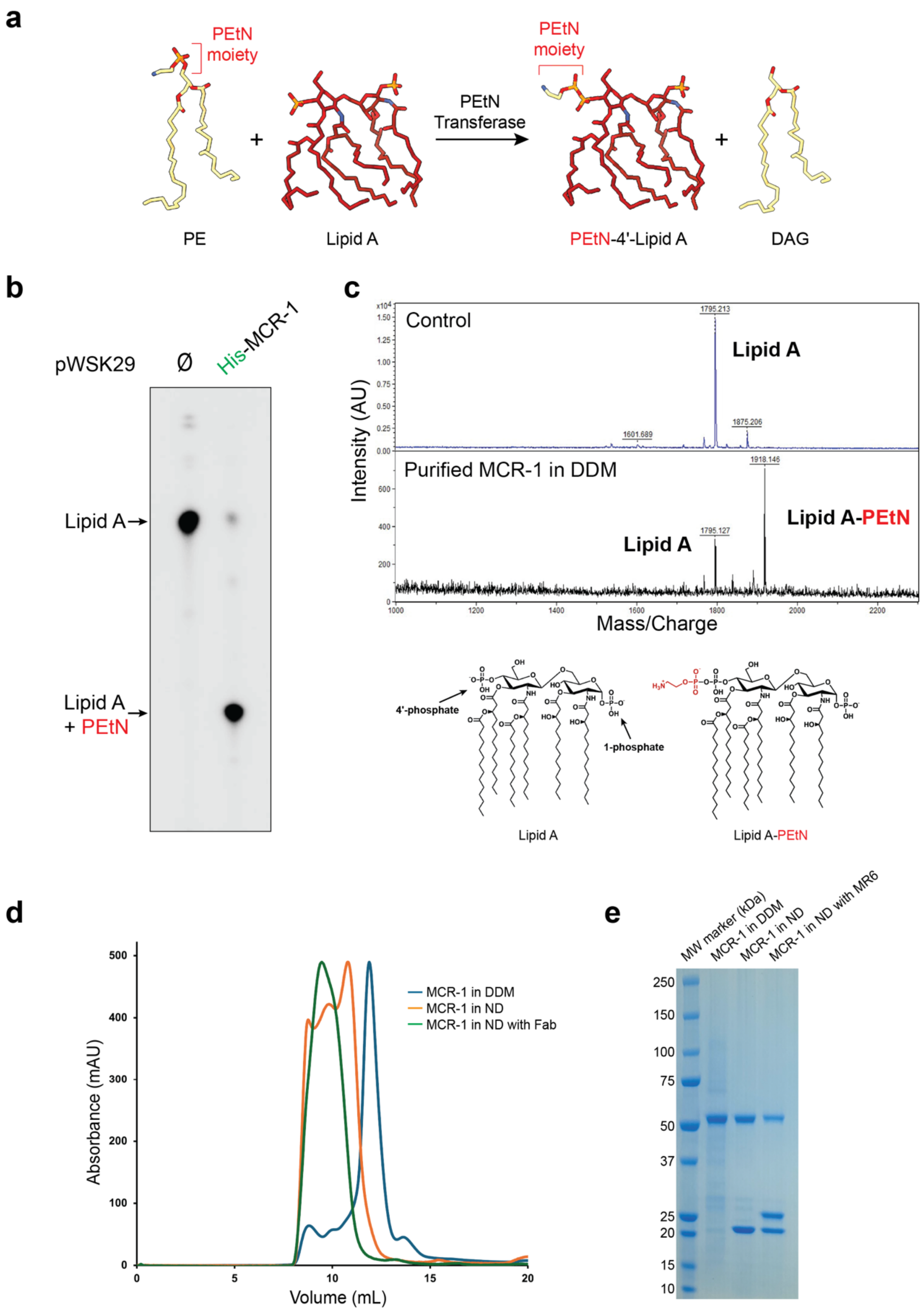

Supplementary Figure 1. Functional validation of MCR-1 and preparation of a nanodisc-reconstituted MCR-1–Fab complex for structural analysis. a, Atomic representation of PEtN

transferase-mediated chemical modification of lipid A. PE, lipid A, PEtN-4'-lipid A, and DAG are shown in stick format and colored as in Fig. 1d. **b**, A TLC plate showing the migration of lipid A isolated from W3110 (Δ*lpxT*, Δ*eptA*) cells grown in LB broth and transformed with empty vector (pWSK29) vector and His-tagged MCR-1. Protein expression was induced with 10 μM IPTG. Major ^{32}P-labeled lipid A species are indicated with an arrow and labeled to the left. **c**, MALDI-TOF analysis of lipid A isolated from control W3110 (Δ*lpxT*, Δ*eptA*) cells, on top, and from DDM-solubilized MCR-1 protein, on bottom. Below, the chemical structures of two species of lipid A – with and without PEtN modification. **d**, Size-exclusion chromatography elution profiles of purified MCR-1 in detergent (blue), incorporated into a nanodisc (orange), and incorporated into a nanodisc with Fab (MR6) bound (green). **e**, SDS-PAGE gel of MCR-1 purification. First lane is MCR-1 purified in DDM, second lane is MCR-1 reconstituted into nanodisc (MSP1D1 and POPG), and third lane is MCR-1 reconstituted into nanodisc (MSP1D1 and POPG) with Fab ((MR6) bound.

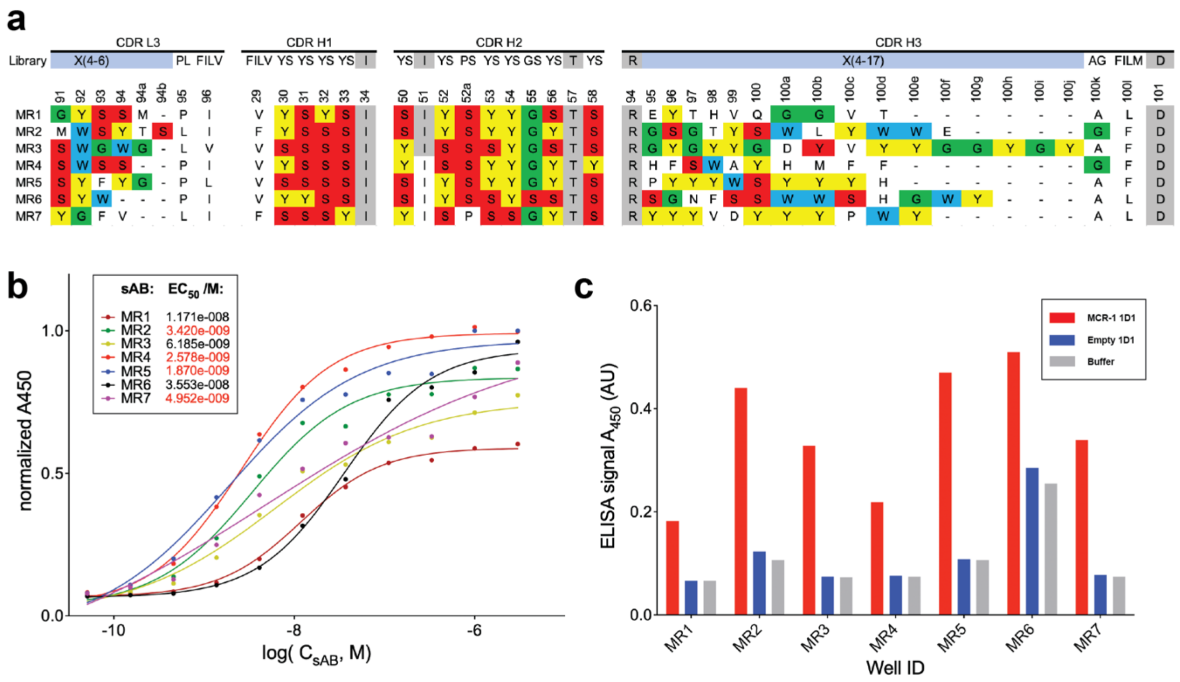

Supplementary Figure 2. Identification of MCR-1–specific Fabs. **a**, Complementary-determining region (CDR) sequences of unique synthetic antigen binders (sABs) from biopanning against MCR-1 in MSP1D1 nanodisc. sABs were selected following multiple rounds of phage display starting from Fab Library E[1,2]. Enriched YSGW residues are highlighted by colored boxes (yellow, red, green, and blue, respectively). YSGW residues have been previously shown to play dominant roles in highly specific and high affinity antigen recognition[3]. **b**, Multi-point sAB ELISA: EC_{50} estimation for purified sAB binding to MCR-1 incorporated into MSP1D1 nanodisc, showing high affinity binding of MR2 (green), MR4 (orange), MR5 (blue), and MR7 (purple), and modest affinity binding of MR1 (red), MR3 (yellow), and MR6 (black). EC_{50} values represent the mean of three independent experiments +/- standard error (n = 3). **c**, Single-point ELISA measuring the binding of phage-displayed sABs to MCR-1 in MSP1D1 nanodisc (red), empty nanodisc (blue), or buffer (empty wells, gray). ELISA signal measured at 450 nm absorbance.

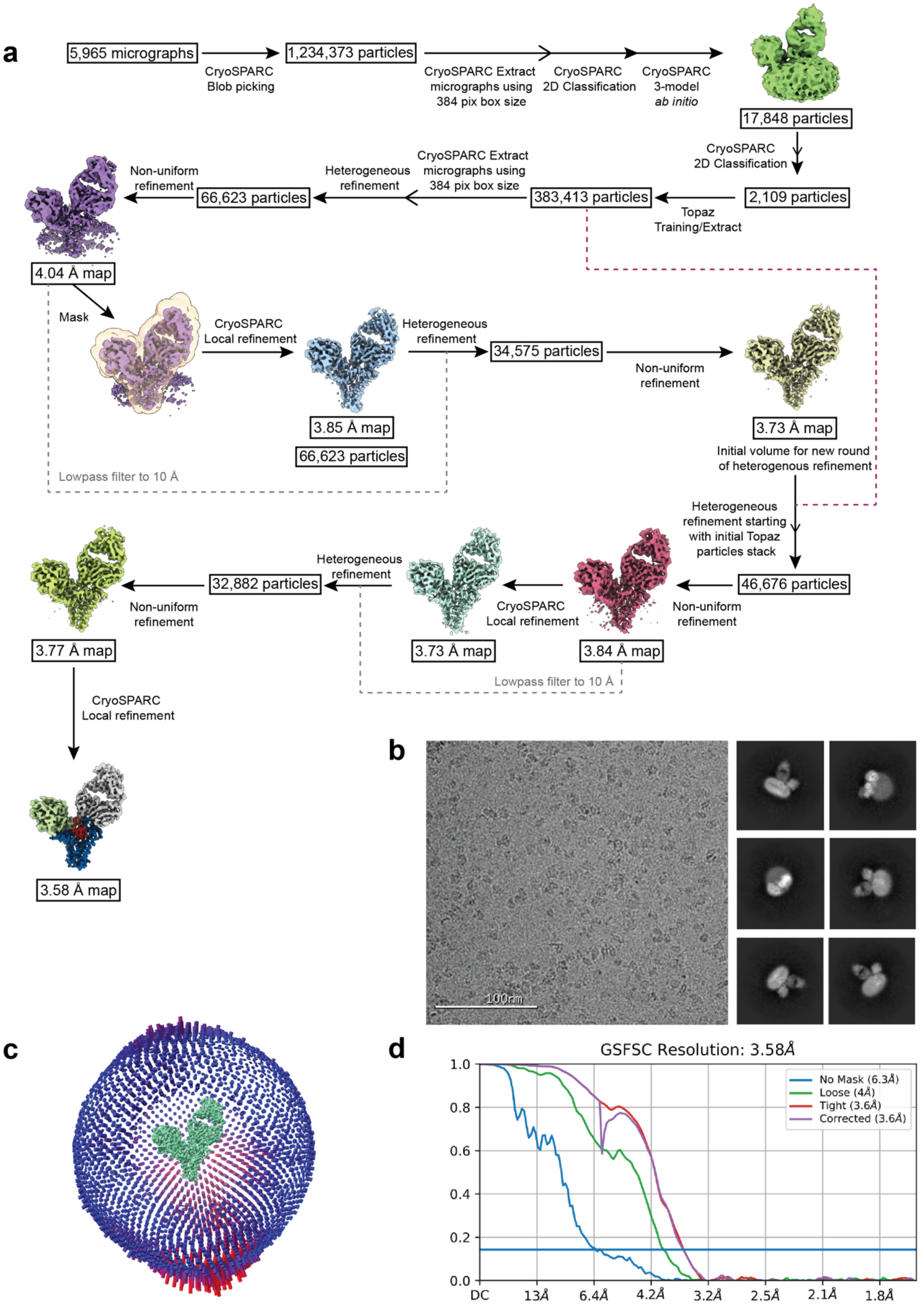
a
5,965 micrographs
CryoSPARC Blob picking
1,234,373 particles
CryoSPARC Extract micrographs using 384 pix box size
CryoSPARC 2D Classification
CryoSPARC 3-model ab initio
17,848 particles
CryoSPARC 2D Classification
2,109 particles
Topaz Training/Extract
383,413 particles
CryoSPARC Extract micrographs using 384 pix box size
Heterogeneous refinement
66,623 particles
Non-uniform refinement
4.04 Å map
Mask
CryoSPARC Local refinement
3.85 Å map
66,623 particles
Heterogeneous refinement
34,575 particles
Non-uniform refinement
3.73 Å map
Initial volume for new round of heterogenous refinement
Lowpass filter to 10 Å
Heterogeneous refinement starting with initial Topaz particles stack
46,676 particles
Non-uniform refinement
3.84 Å map
CryoSPARC Local refinement
3.73 Å map
Lowpass filter to 10 Å
Heterogeneous refinement
32,882 particles
Non-uniform refinement
3.77 Å map
CryoSPARC Local refinement
3.58 Å map
b
100nm
c
d
GSFSC Resolution: 3.58Å
No Mask (6.3Å)
Loose (4Å)
Tight (3.6Å)
Corrected (3.6Å)
1.0
0.8
0.6
0.4
0.2
0.0
DC
13Å
6.4Å
4.2Å
3.2Å
2.5Å
2.1Å
1.8Å

Supplementary Figure 3. Cryo-EM analysis of the MCR-1 –Fab complex in nanodisc. a, Flow chart outlining cryo-EM data processing and refinement performed to obtain a structure of nanodisc-reconstituted MCR-1 with the Fab MR6. Blob picker in cryoSPARC v.4.0.1[4] was used to pick 1,234,373 particles, which were subjected to 2D classification. One round of *ab initio* reconstruction was performed and the best class was selected. Further 2D classification was carried out on this particle stack, after which a subset of particles was selected, curated, and used as input for Topaz training, particle picking, and extraction among the full set of exposures, resulting in a stack of 383,413 particles. Heterogeneous refinement was carried out on this particle stack and repeated three times using the particle output associated with the volumes from the previous heterogeneous refinement as input for the next round. From this final heterogeneous refinement, particles from the best class were selected and re-extracted and subjected to non-uniform refinement, resulting in a 4.04 Å reconstruction. Using a mask covering MCR-1 and the Fab, local refinement was performed, resulting in a 3.85 Å density map. The particles were further sorted by two class heterogeneous refinement using the map from the 3.85 Å local refinement and a 10 Å lowpass filtered map as inputs. Particles associated with the lowpass filtered volume were excluded, with the remaining particle stack then subjected to non-uniform refinement, yielding a map with a resolution of 3.73 Å. Multiple additional rounds of heterogeneous refinement were performed, using the particle-stack from Topaz picking as input along with three old *ab* initio volumes and the 3.73 Å volume as the initial volumes for the new round of heterogenous refinement. Particles from the best class were selected and subjected to non-uniform and local refinement, generating a 3.73 Å map. The previously described two-class heterogenous refinement, using a 10 Å lowpass filtered map as one input volume, was performed, yielding a particle stack that was subsequently used as input for an additional non-uniform refinement to

produce a 3.77 Å density map. Local refinement was performed using the previous mask, resulting in a final resolution of 3.58 Å. **b**, On the left, representative micrograph (-2.02 μm defocus). On right, representative 2D class averages from CryoSPARC 2D classification[5]. **c**, Euler angle distribution of all particles used in the final map reconstruction. Final map is shown in green. Each orientation is represented by a cylinder, with the height of each cylinder and color (from blue to red) proportional to the number of particles for that specific region. **d**, Fourier shell correlation (FSC) curves for the PE- and KLA-bound MCR-1–MR6 complex.

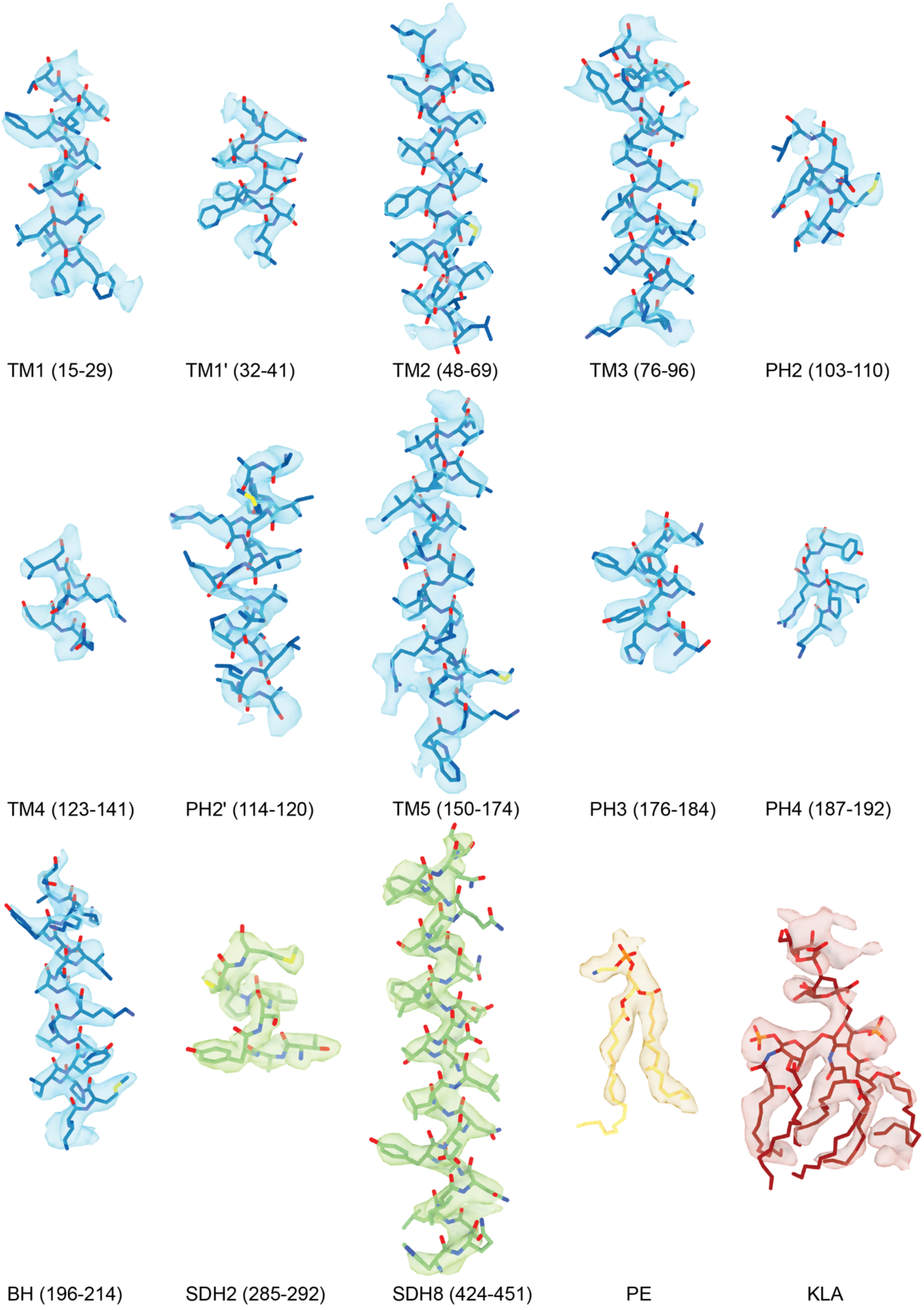
TM1 (15-29)
TM1' (32-41)
TM2 (48-69)
TM3 (76-96)
PH2 (103-110)
TM4 (123-141)
PH2' (114-120)
TM5 (150-174)
PH3 (176-184)
PH4 (187-192)
BH (196-214)
SDH2 (285-292)
SDH8 (424-451)
PE
KLA

Supplementary Figure 4. Fit of cryo-EM density with model. Cryo-EM densities (semi-transparent surfaces) are superimposed on TM helices, the BH, PH2, PH2', PH3, PH4, and select helices from the PD of the MCR-1 model, along with PE and KLA ligands. The model is rendered as side chain and colored as in Fig. 1d. PE (yellow) and KLA (red) are shown as sticks.

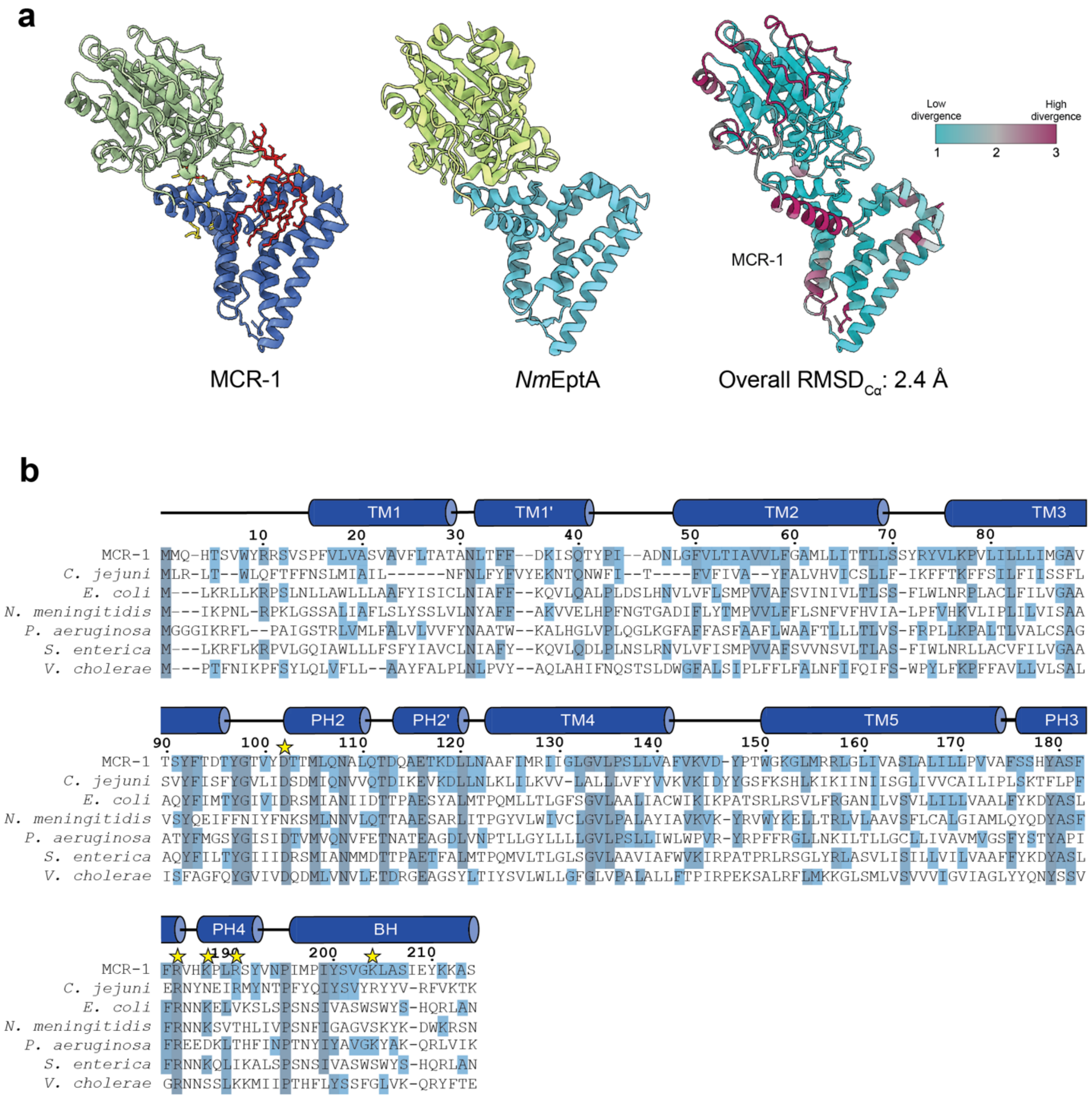

Supplementary Figure 5. Comparison of MCR-1 and *Nm*EptA structures. a, On left, the PE- and KLA-bound MCR-1 cryo-EM structure (colored as in Fig. 1d) is shown in cartoon form with PE and KLA shown as sticks; center, the crystal structure of apo *Nm*EptA (PDB ID: 5FGN[6]) is shown in cartoon form, with the PD colored a bright green and the TM domain colored light blue; on right, the MCR-1 model is shown again, without PE and KLA substrates, and colored locally by Cα RMSD relative to the *Nm*EptA crystal structure, with cyan and purple indicating low and

high divergence, respectively. **b**, Sequence alignment and secondary structure of the TM domain of MCR-1 and EptA from *C. jejuni* (*Cj*EptC), *E. coli* (*Ec*EptA), *N. meningitidis* (*Nm*EptA), *P. aeruginosa* (*Pa*EptA), *S. enterica* (*Se*EptA), and *V. cholerae* (*Vc*EptA). Conserved residues are highlighted in shades of blue, with darker shading indicative of greater conservation to MCR-1. Yellow stars above the sequence denote functionally relevant MCR-1 residues near the KLA binding site.

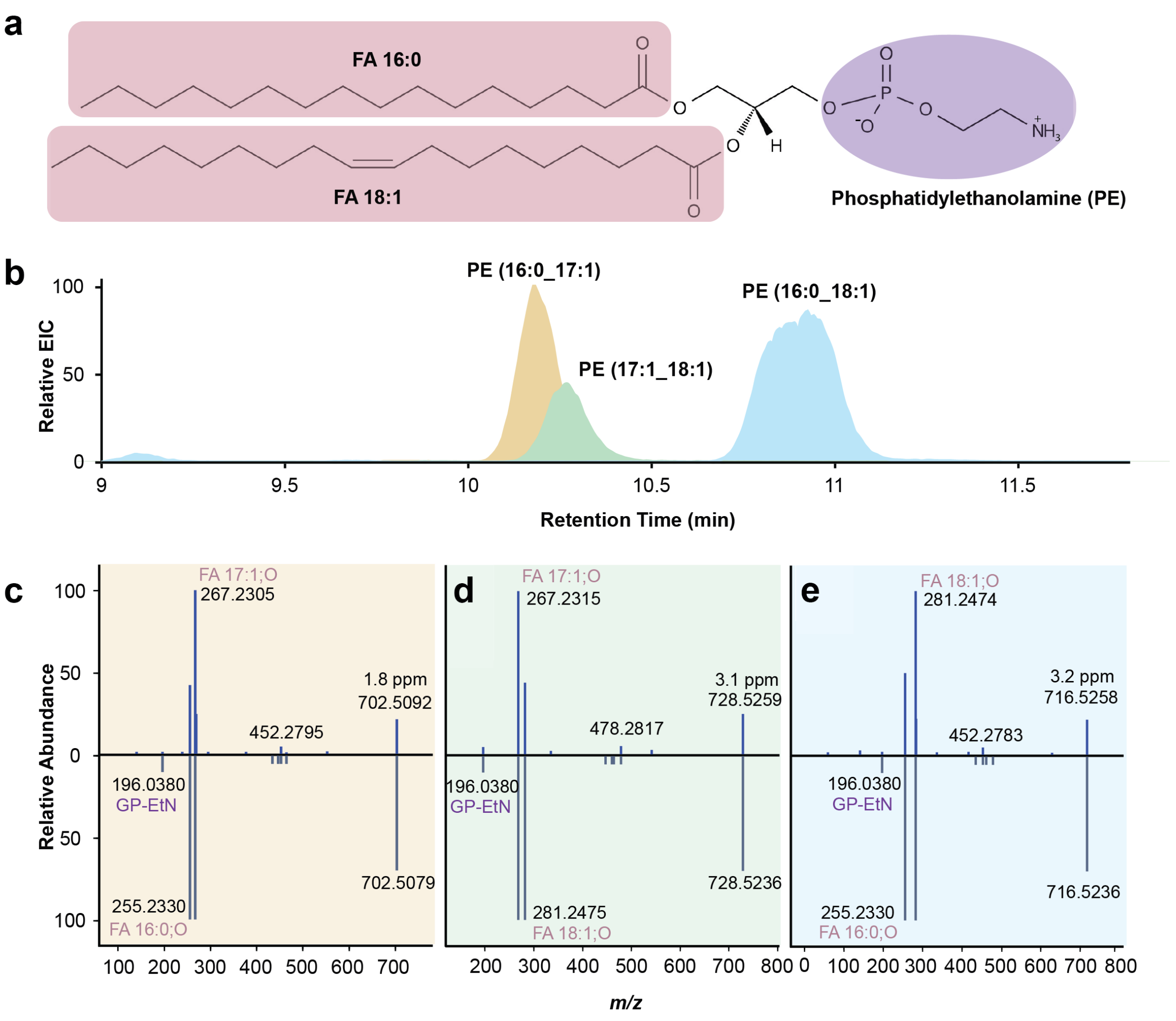

Supplementary Figure 6. Identification of PE from purified MCR-1 using LC-MS. Annotation of PE lipids detected in MCR-1 nanodiscs. Both PE and cyclopropyl-PE were observed. **a**, Chemical structure of PE (16:0_18:1). **b**, Extracted ion chromatogram (EIC) of annotated PE species. **c**–**e**, MS/MS fragmentation spectra (*top*) of two cyclopropyl-PE species (16:0_17:1 and 17:1_18:1), and PE (16:0_18:1), shown alongside their corresponding reference library spectra (*bottom*) for comparison.

a

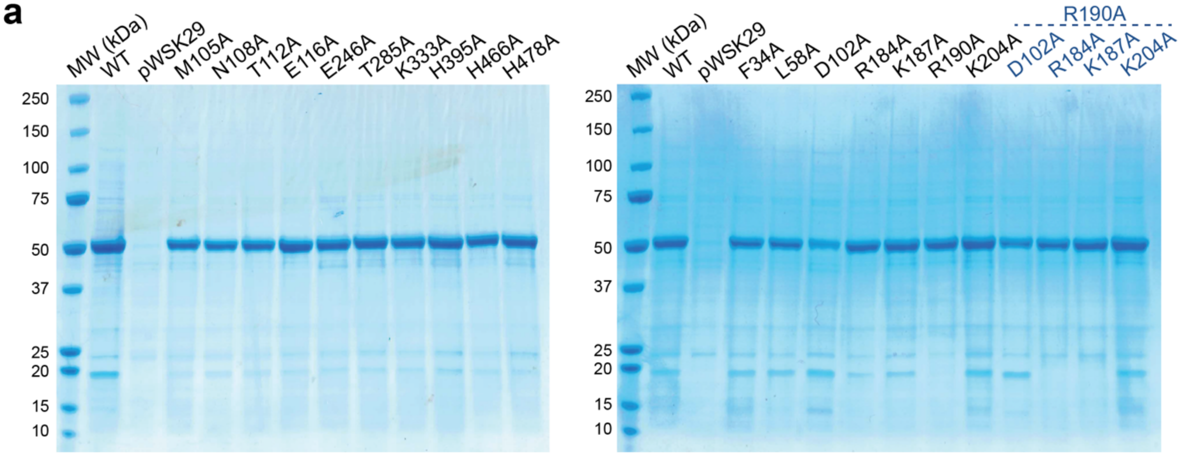

b

Mutations targeting **PE binding site**

	WT	pWSK29	M105A	N108A	T112A	E116A	E246A	T285A	K333A	H395A	H466A	H478A
Lipid A	13	100	57	26	41	99	100	100	63	100	99	100
Lipid A + PEtN	87	0	43	74	59	1	0	0	37	0	1	0

Mutations targeting **Lipid A binding site**

	WT	pWSK29	F34A	L58A	D102A	R184A	K187A	R190A	K204A	D102A/R190A	R184A/R190A	K187A/R190A	R190A/K204A
Lipid A	15	100	26	18	34	33	15	72	16	79	75	94	82
Lipid A + PEtN	85	0	74	82	66	67	85	28	84	21	25	6	18

c

MCR-1 Protein	% PEtN-Lipid A	MIC PMB (µg/mL)
None	0	<0.064
WT	87	3.0
M105A	43	<0.064
T112A	59	<0.064
K187A	85	2.0
R190A	28	<0.064
E246A	0	<0.064
K333A	37	<0.064
H395A	0	<0.064
K187A/R190A	6	<0.064

Supplementary Figure 7. Analysis of MCR-1 mutant expression and activity. a, SDS-PAGE gel of MCR-1 mutants targeting the PE and KLA binding sites, purified to verify expression. **b**, Tables displaying the percent of unmodified and PEtN-modified lipid A, as determined by differences in migration on a TLC plate, isolated from W3110 (Δ*lpxT*, Δ*eptA*) cells expressing the indicated constructs (MCR-1 WT, empty pWSK29 vector, and MCR-1 mutants). **c**, Table

displaying the MIC of PMB for select mutants, along with the percent of PEtN-modified lipid A from cells expressing the indicated mutant, taken from **b**, for comparison.

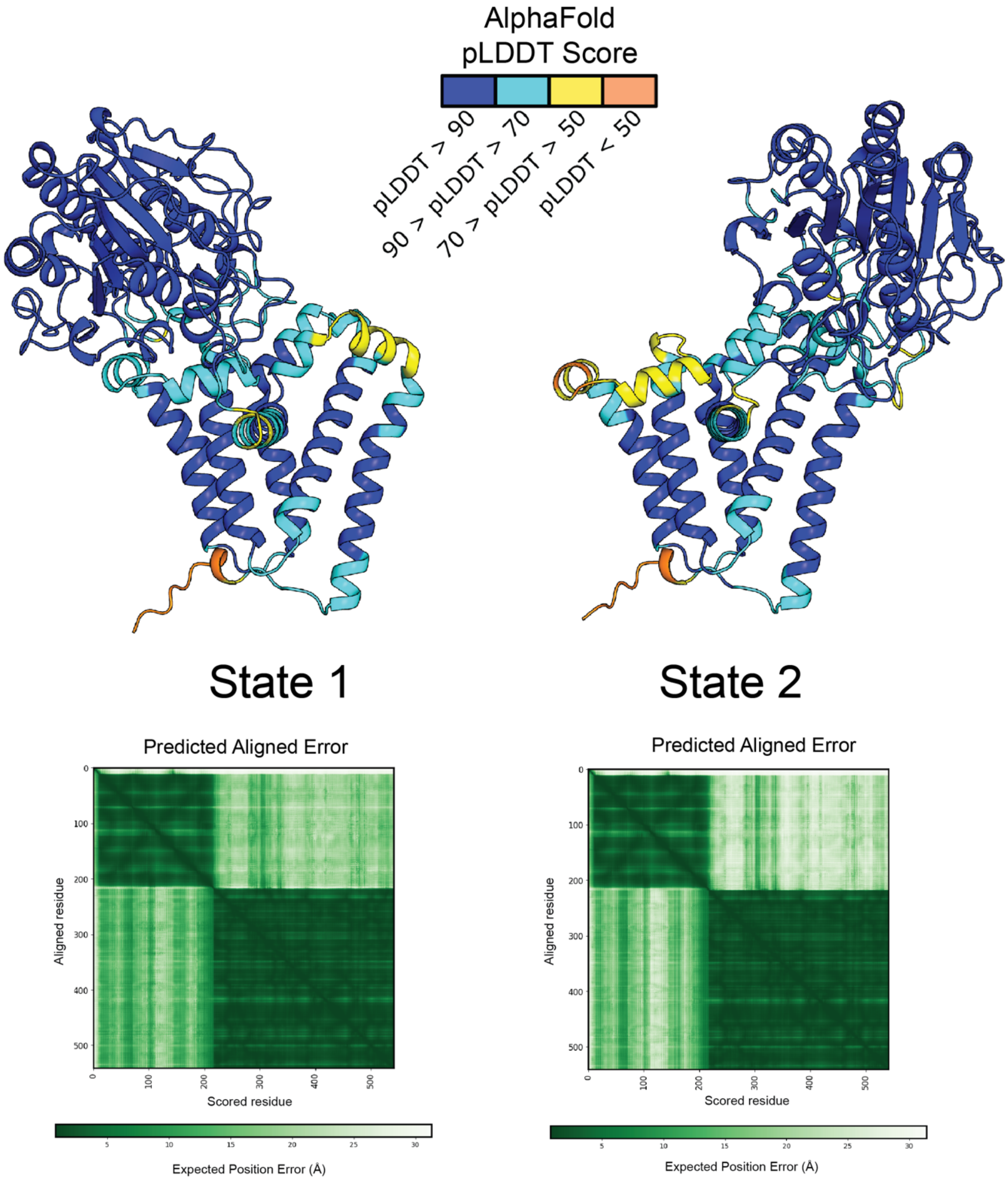

Supplementary Figure 8. AlphaFold models. On the left, an AlphaFold model of MCR-1 is shown in a conformation very similar to that observed in our cryo-EM structure (State 1). On the right, another AlphaFold model predicts a second major conformation, with the PD rotated along the BH, positioned above the KLA binding site between PH3 and PH4 (State 2). Below each model

are respective predicted aligned error (PAE) heatmap plots, presented on a gradient scale from green to white (0 to 30 Å).

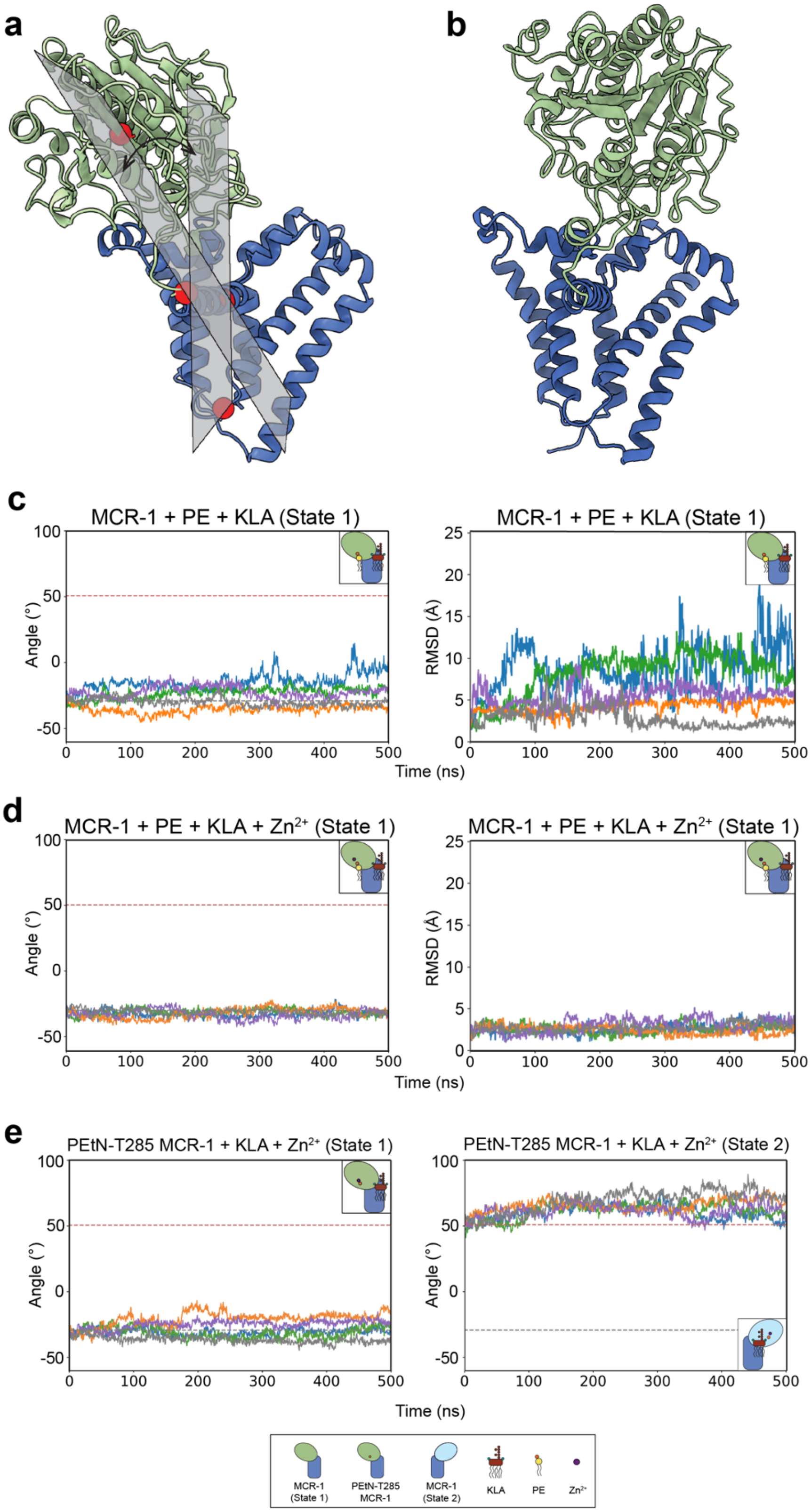
a
b
c
MCR-1 + PE + KLA (State 1)
Angle (°)
RMSD (Å)
Time (ns)
d
MCR-1 + PE + KLA + Zn2+ (State 1)
e
PEtN-T285 MCR-1 + KLA + Zn2+ (State 1)
PEtN-T285 MCR-1 + KLA + Zn2+ (State 2)
MCR-1 (State 1)
PEtN-T285 MCR-1
MCR-1 (State 2)
KLA
PE
Zn2+

Supplementary Figure 9. MCR-1 model with planes of reference for MD simulations, and additional ligand-bound simulations. a, The MCR-1 cryo-EM structure, shown in cartoon form without PE and KLA and colored as in Fig. 1d, with small red spheres replacing residues in the TM domain (Y72), the BH (P195 and A215), and the PD (V243). Two planes are drawn through the structure: one at an angle, running through V243 and intersecting P195 and A215 in the BH, the other oriented close 180° and running through Y72 and intersecting P195 and A215 in the BH. Above the BH is an arrow indicating the angle between the two planes. A negative angle (~25°) is depicted by the State 1 conformation. **b**, Transient conformation adopted by MCR-1 during a partial transition from State 1 to State 2. **c**, On left, five repeats of atomistic MD simulations over 500 ns performed with PE- and KLA-bound MCR-1 in State 1. On right, a time trace of PE RMSD without Zn^{2+}. **d**, On left, five repeats of atomistic MD simulations over 500 ns performed with PE- and KLA-bound MCR-1 in State 1 with Zn^{2+}. On right, a time trace of PE RMSD with Zn^{2+}. **e**, On left, five repeats of atomistic MD simulations over 500 ns performed with PEtN-T285 MCR-1 in State 1 with Zn^{2+}. On right, five repeats of atomistic MD simulations over 500 ns performed with PEtN-T285 MCR-1 in State 2 with Zn^{2+}. For each group of simulations, MCR-1, PE, and KLA are shown as small cartoons colored as in Fig. 1a, with the exception of the cartoon for MCR-1 in State 2 which is colored as in Fig. 3, and depicted in the top right corner for the State 1 simulations and the bottom right corner for the State 2 simulations. Zn^{2+} is depicted as a small purple sphere. The simulations are colored gray, green, orange, purple, and blue, respectively. The dashed gray and red lines correspond to the angles for the State 1 and State 2 conformations, respectively. Source data are provided as a Source Data file.

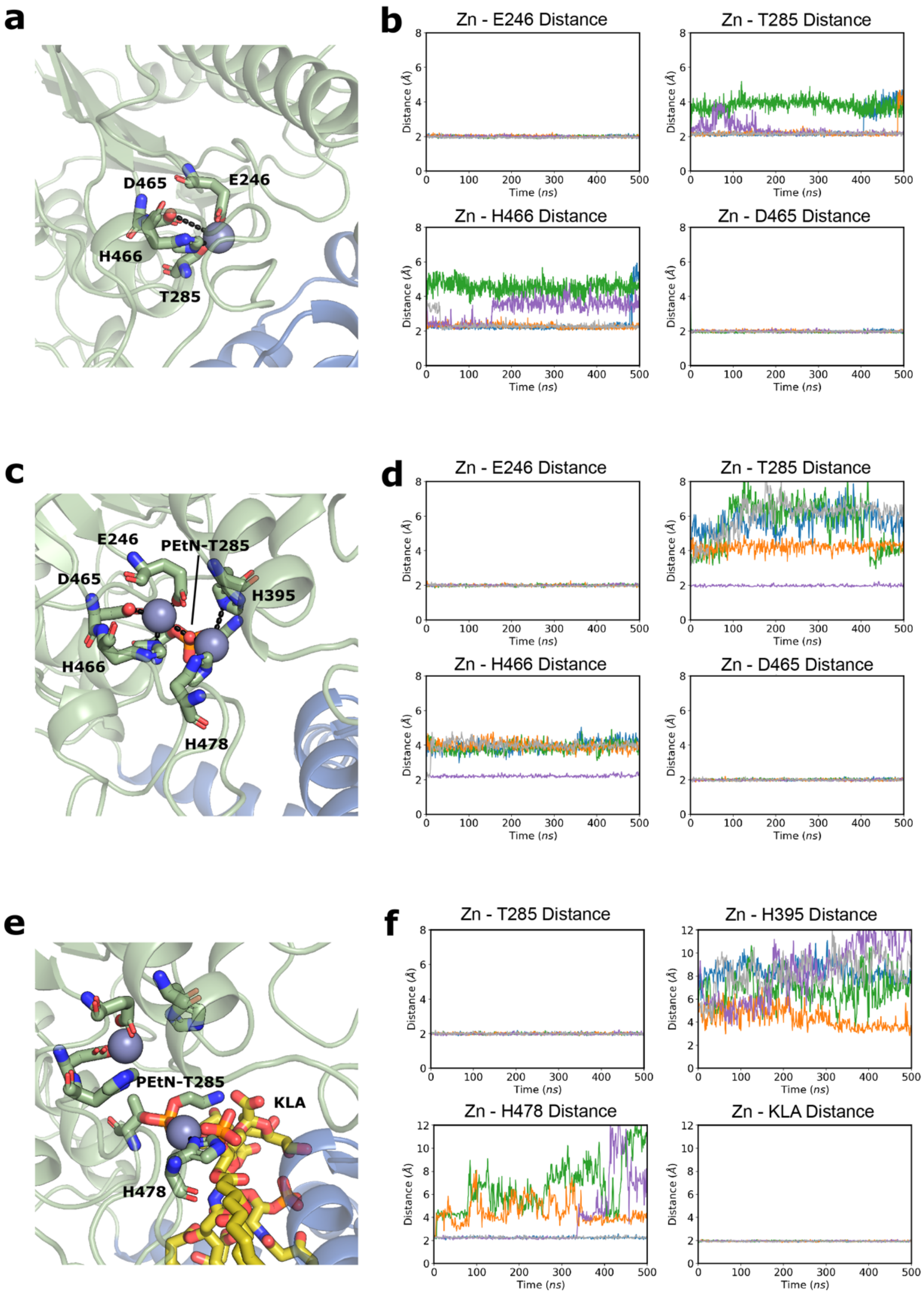

Supplementary Figure 10. Zinc coordination. a, A zoomed in panel depicting the Zn^{2+} ion binding site in the State 1 conformation. The ion was modelled based on the *Nm*EptA structure

(PDB ID: 5FGN)[6] and is coordinated by the residues E246, T285, D465 and H466. **b**, Time traces of the distance between Zn^{2+} and the coordinating residues E246 (top left), T285 (top right), H466 (bottom left) and D465 (bottom right). **c**, A zoomed in view showing the ions Zn^{2+} binding sites in the State 2 conformation. The ions coordinates were modelled using the di-zinc MCR-1 structure (PDB ID: 5LRM)[7]. The first Zn^{2+} ion is coordinated by the same residues as in State 1. The second ion is coordinated by the residues T285, H395 and H478. **d**, Time traces of the distance between the first Zn^{2+} ion and the residues E246 (top left), T285 (top right), H466 (bottom left) and D465 (bottom right). **e**, Final frame of one of the MD simulation repeats showing the repositioning of the second Zn^{2+} ion and its interaction with KLA. **f**, Time traces of the distance between the second Zn^{2+} ion and the residues T285 (top left), H395 (top right), H478 (bottom left) and KLA (bottom right). In our simulations, the second Zn^{2+} ion is primarily coordinated by T285 and a nearby KLA phosphate group.

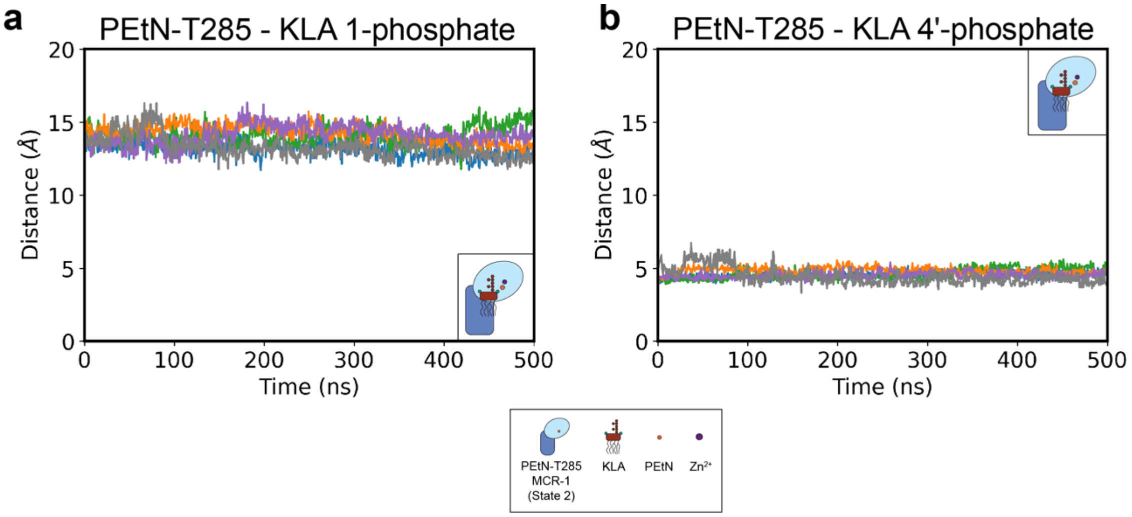

Supplementary Figure 11. PEtN-T285 – KLA distance. Time trace of the distance between the phosphorus atom from PEtN-T285 MCR-1 in State 2 and the phosphorus atom from the KLA 1-phosphate group, **a**, or the KLA 4'-phosphate group, **b**. PEtN-T285 MCR-1, PEtN, Zn^{2+}, and KLA are shown as small cartoons colored as in Supplementary Fig. 9. Source data are provided as a Source Data file.

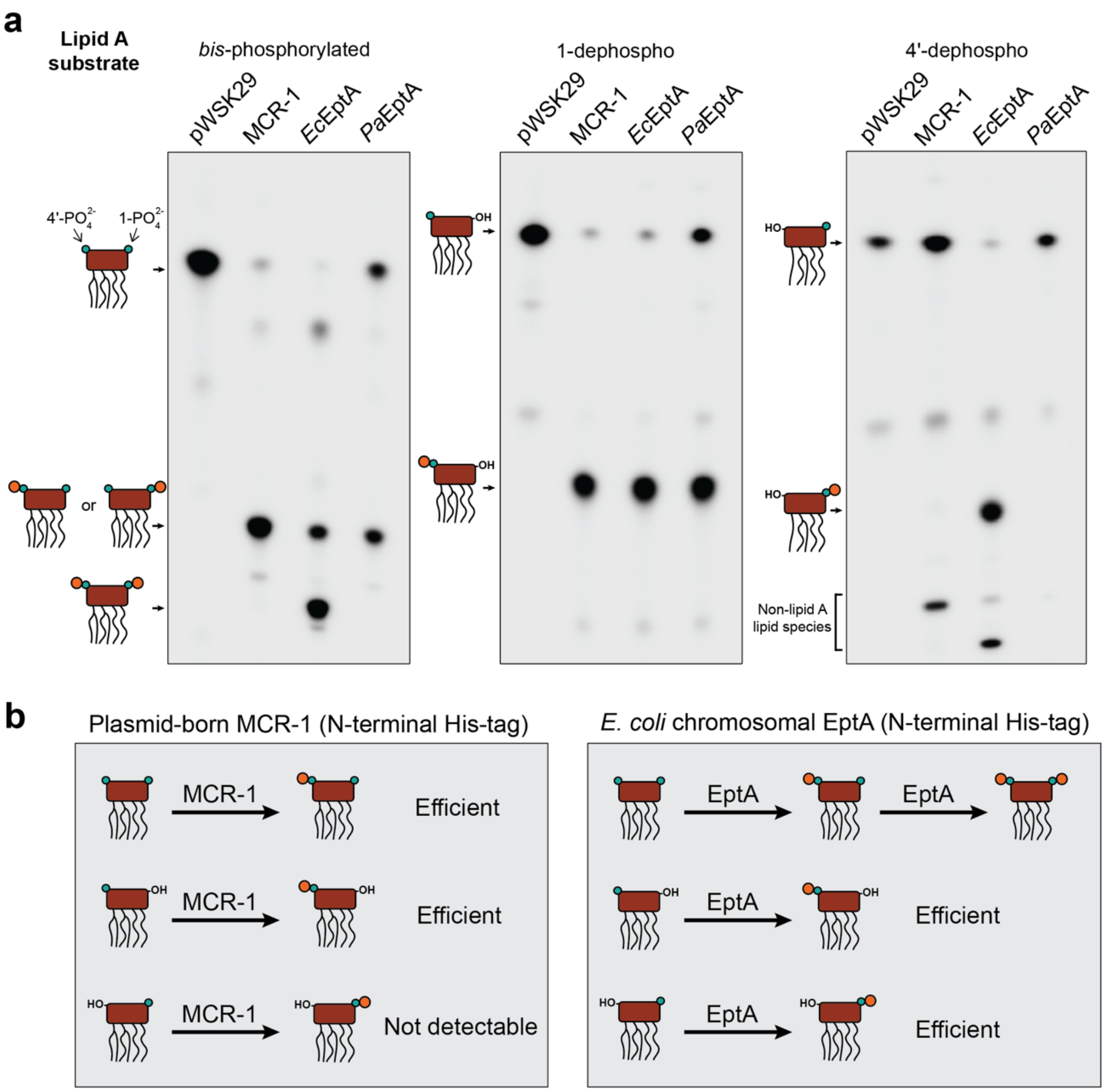

Supplementary Figure 12. Comparison of preferential sites of lipid A modification between MCR-1 and *Ec*EptA. a, On left, a TLC plate showing the migration of lipid A isolated from W3110 (Δ*lpxT*, Δ*eptA*) cells grown in LB broth and transformed with empty pWSK29 vector, MCR-1, *Ec*EptA, and *Pa*EptA as a positive control (previously shown to selectively modify the 4'-phosphate[8]. The genetic background is nearly 100% *bis*-phosphorylated hexa-acylated lipid A. Center, a TLC plate showing the migration of lipid A isolated from W3110 (*lpxT*::*lpxE*, Δ*eptA*) cells grown in LB broth and transformed with empty vector (pWSK29), MCR-1, *Ec*EptA, and

*Pa*EptA. The genetic background is nearly 100% 1-dephosphorylated (LpxE-modified) lipid A. On right, a TLC plate showing the migration of lipid A isolated from W3110 (Δ*lpxT*, Δ*eptA*, Δ*pagP*, *lpxM*::*lpxF*) cells grown in LB broth and transformed with pWSK29, MCR-1, *Ec*EptA, and *Pa*EptA. The genetic background is nearly 100% 4'-dephosphorylated (LpxF-modified) lipid A. Protein expression was induced with 10 μM IPTG. Major ^{32}P-labeled lipid A species are indicated with an arrow and shown in simplified cartoon form on the left side of the TLC images, colored as in Fig. 1a. **b**, Graphic outlining the outcomes between N-terminally His-tagged MCR-1 and N-terminally His-tagged *Ec*EptA when expressed in genetic backgrounds producing *bis*-phosphorylated, 1-dephosphorylated, and 4'-dephosphorylated lipid A species, respectively. On left, MCR-1 can efficiently perform PEtN modification at a single phosphate on *bis*-phosphorylated lipid A or on the 4'-phosphate group of 1-dephosphorylated lipid A. MCR-1 cannot efficiently modify the 1-phosphate group on 4'-dephosphorylated lipid A. On right, *Ec*EptA is able to efficiently modify *bis*-phosphorylated lipid A at a single site as well as both phosphate groups, and can also efficiently modify the 4'-phosphate on 1-dephosphorylated lipid A and the 1-phosphate on 4'-dephosphorylated lipid A.

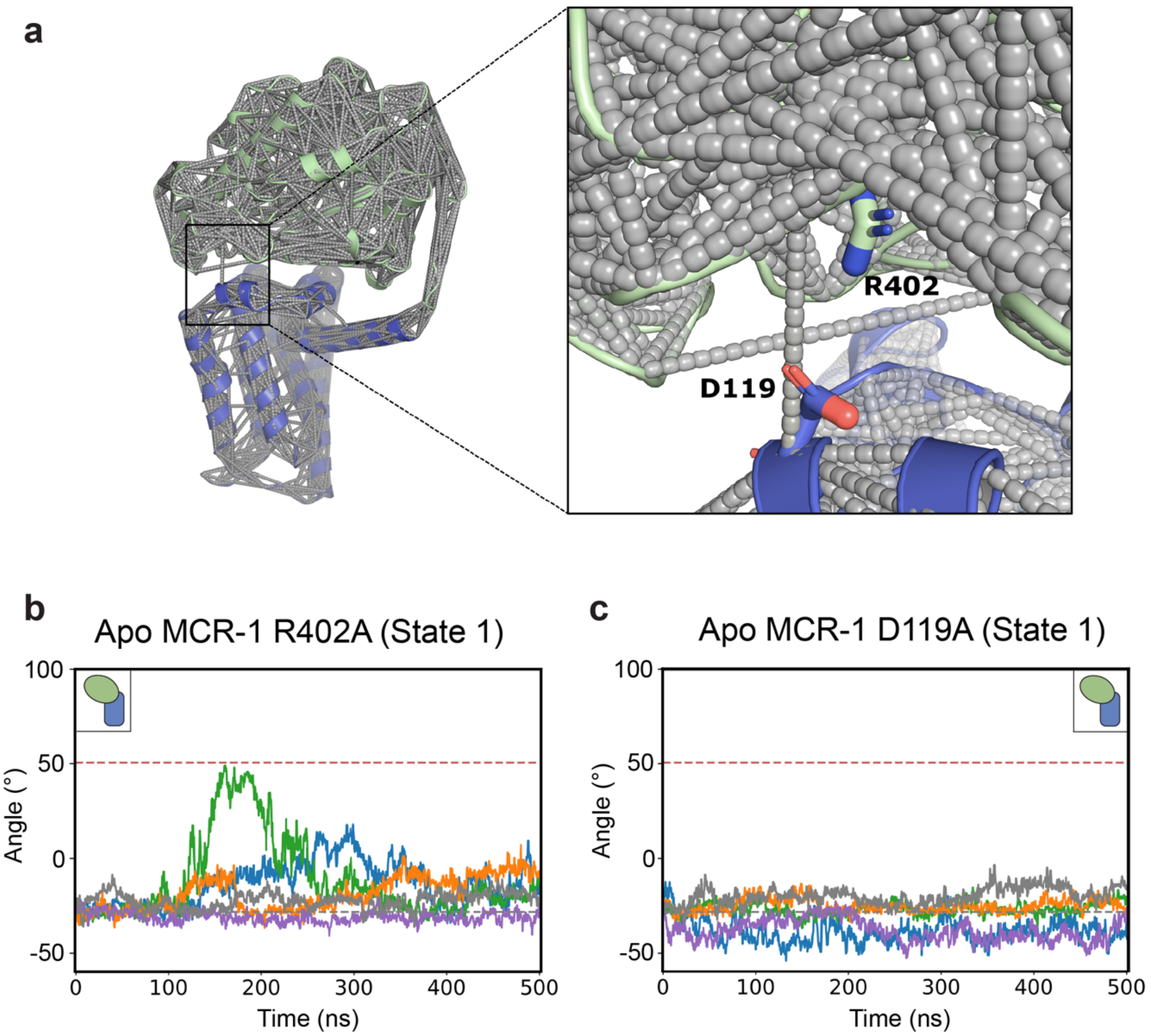

Supplementary Figure 13. Sequence coevolution analysis. a, On left, a structural representation of the predicted coevolutionary residue contacts for MCR-1. On right, a zoomed in view highlighting the single predicted inter-domain contact. **b**, Five repeats of atomistic MD simulations over 500 ns performed with the apo R402A MCR-1 mutant in the State 1 conformation, depicted in the top right corner as a cartoon colored as in Fig. 1a. A full transition from the State 1 conformation to the State 2 conformation is observed for the R402A MCR-1 mutant (green curve). **c**, Five repeats of atomistic MD simulations over 500 ns performed with the apo D119A MCR-1 mutant in the State 1 conformation, depicted in the top left corner as a cartoon colored as in Fig.

1a. The dashed gray and red lines correspond to the angles for the State 1 and State 2 conformations, respectively. Source data are provided as a Source Data file.

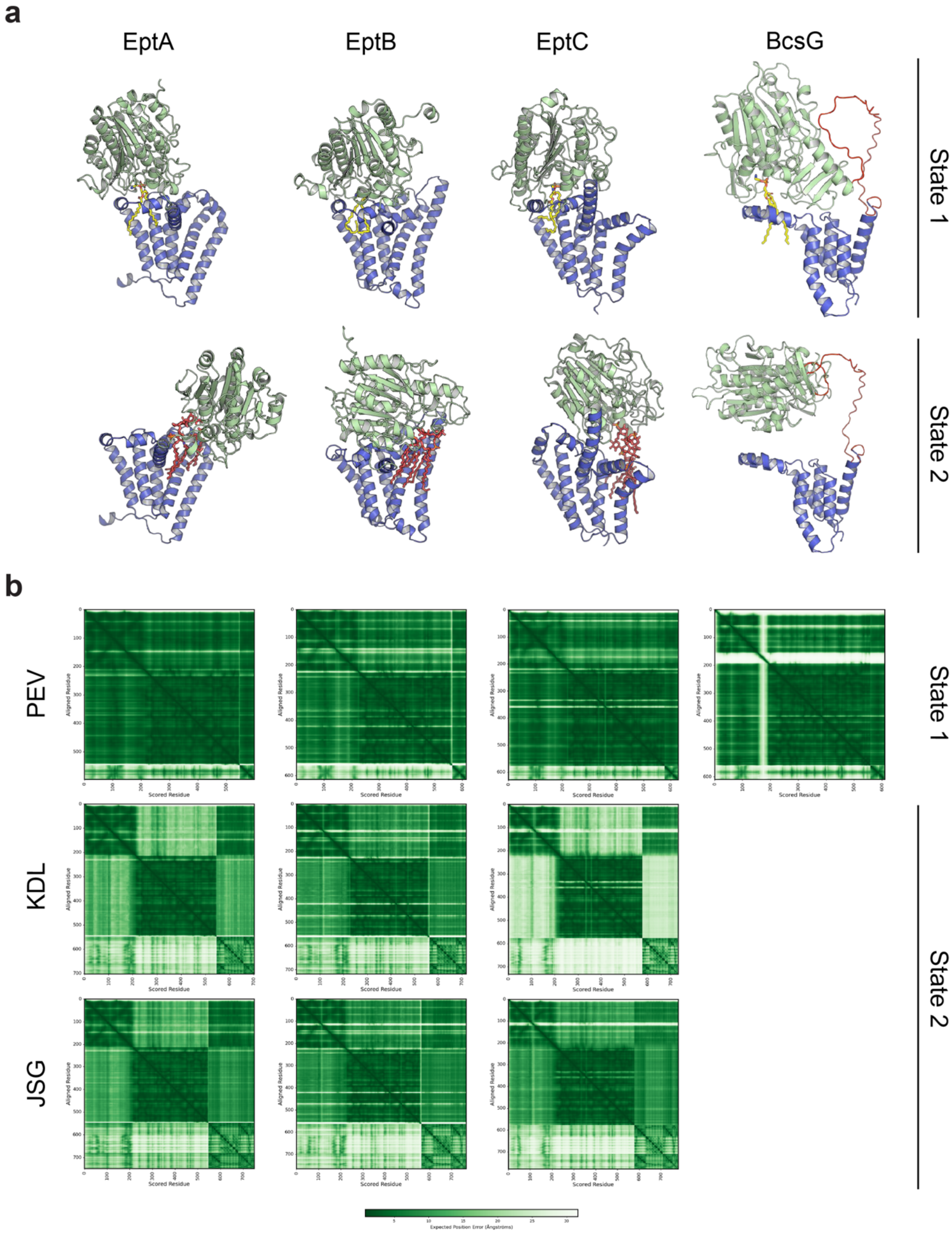

Supplementary Figure 14. Mechanistic similarities between MCR-1 and other phosphoform transferases. a, AlphaFold3 models depicting the State 1 and State 2 conformations of EptA,

EptB, EptC, and BcsG from *E. coli* K12. The structures are colored similarly to MCR-1 in Fig. 1, with the soluble domain green and TM domain blue. PE and lipid A are depicted in stick format and colored as in Fig. 1. **b**, AlphaFold3 PAE plots for EptA, EptB, EptC, and BcsG are presented on a gradient scale from green to white (0 to 30 Å), with bound CCD coordinates for PEV (PE), KDL (KLA), or JSG (Lipid A-core oligosaccharide). In the case of BcsG, only PEV binding is modeled. Whenever PEV is present, models corresponding to State 1 were generated. In contrast, when LPS molecules were used, AlphaFold3 returned models representing State 2. While MCR-1 and EptA display similar state characteristics, the second state of EptB and EptC appears to occupy an intermediate position, potentially facilitating the modification of Kdo and core oligosaccharide heptose sugars, respectively. Source data are provided as a Source Data file.

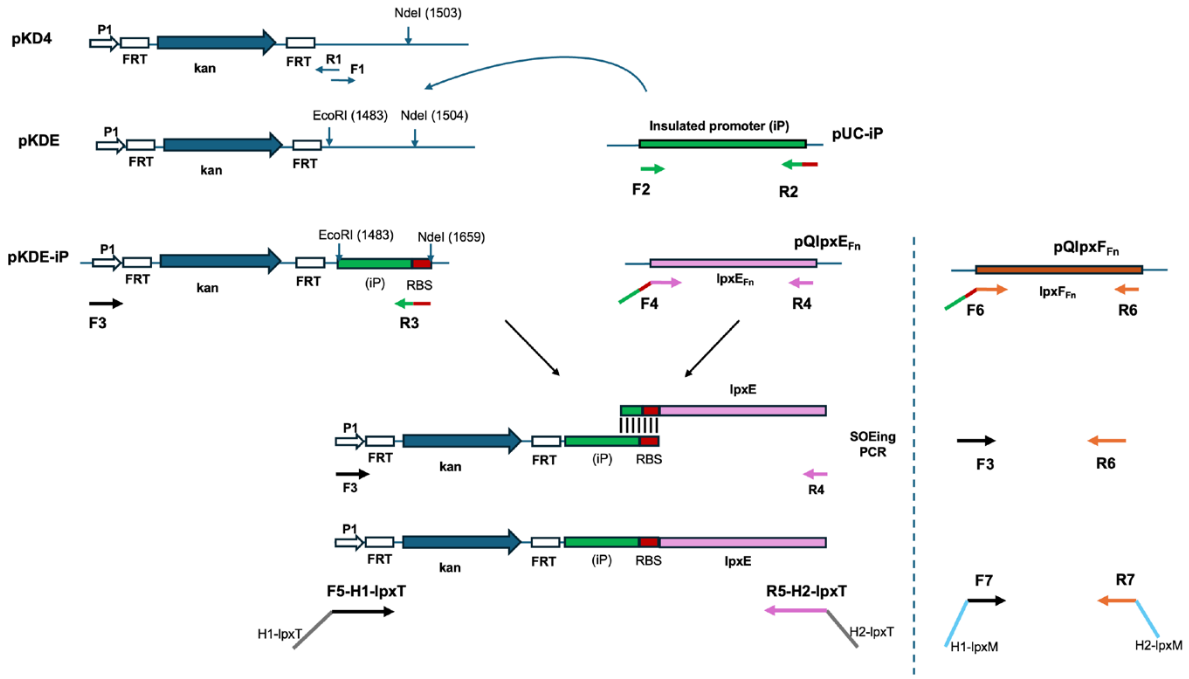

Supplementary Figure 15. Schematic showing the genetic engineering of strains to produce either 1-dephosphorylated or 4'-dephosphorylated lipid A species. The schematic shows how an insulated promoter (iP in figure) was introduced into plasmid pKD4 to generate plasmid pKDE-iP. This plasmid was used in subsequent assembly PCR steps to generate PCR products for recombineering to insert either *lpxE* or *lpxF* under the control of an insulated promoter into the *E. coli* chromosomal. See methods for further details.

Supplementary Table 1. Cryo-EM data collection statistics.

	MCR-1–MR6 (PE & KLA bound)<br>(PDB – 9NWW)<br>(EMD – 49896)
**Data collection and processing**	
Magnification	105,000
Voltage (kV)	300
Electron exposure (e-/Å^2)	58.2
Exposure time (s)	2.5
Dose rate (e-/pixel/s)	16.1
Nominal defocus range (um)	-2.5 to -1
Defocus range (um)	-2.2 to -0.8
Pixel size (Å)	0.83
Symmetry imposed	C1
Number of micrographs	5,965
Initial particle images (no.)	1,234,373
Final particle images (no.)	32,882
Map resolution (Å)	3.58
FSC threshold	0.143
**Refinement**	
Map sharpening *B* factor (Å^2)	86.8
Model composition	6,047
Non-hydrogen atoms	753
Protein residues	532
Ligands	2
R.m.s deviations	
Bond lengths (Å)	0.005
Bond angles (°)	0.753
Validation	
MolProbity score	1.88
Clashscore	8.85
Poor rotamers (%)	0.94
Ramachandran plot	
Favored (%)	93.84
Allowed (%)	4.95
Disallowed (%)	1.20

Supplementary Table 2. Primer sequences for mutagenesis and the amino acid sequence of His-tagged MCR-1.

Mutant	Primer Sequences
F34A	Forward: cgaccgccaatcttaccgcttttgataaaatcagc<br>Reverse: gctgattttatcaaaagcggtaagattggcggtcg
L58A	Forward: gatcgctgtcgtggcctttggcgcgatg<br>Reverse: catcgcgccaaaggccacgacagcgatc
D102A	Forward: gcacggtctatgctacgaccatgctc<br>Reverse: gagcatggtcgtagcatagaccgtgc
M105A	Forward: gtctatgatacgaccgcgctccaaaatgccc<br>Reverse: gggcattttggagcgcggtcgtatcatagac
N108A	Forward: gaccatgctccaagctgccctgcagac<br>Reverse: gtctgcagggcagcttggagcatggtc
T112A	Forward: caaaatgccctgcaggccgaccaagccgagac<br>Reverse: gtctcggcttggtcggcctgcagggcattttg
E116A	Forward: ctgcagaccgaccaagccgcgaccaaggatctgttaaac<br>Reverse: gtttaacagatccttggtcgcggcttggtcggtctgcag
R184A	Forward: cattatgccagtttctttgccgtgcataagccgctgc<br>Reverse: gcagcggcttatgcacggcaaagaaactggcataatg
K187A	Forward: gtttctttcgcgtgcatgcgccgctgcgtagctatg<br>Reverse: catagctacgcagcggcgcatgcacgcgaaagaaac
R190A	Forward: gtgcataagccgctggctagctatgtcaatc<br>Reverse: gattgacatagctagccagcggcttatgcac
K204A	Forward: caatctactcggtgggtgcacttgccagtattgag<br>Reverse: ctcaatactggcaagtgcacccaccgagtagattg
E246A	Forward: cgtcgtcggtgcgacggcacgcg<br>Reverse: cgcgtgccgtcgcaccgacgacg
T285A	Forward: gtgcggcacatcggcggcgtattctg<br>Reverse: cagaatacgccgccgatgtgccgcac
K333A	Forward: gataataattcggactcagcaggcgtgatggataagc<br>Reverse: gcttatccatcagcgctgctgagtccgaattattatc
H395A	Forward: ctgcaccaaatgggcaatgccgggcctgcgtattttaag<br>Reverse: cttaaaatacgcaggcccggcattgcccatttggtgcag
H466A	Forward: ctgtatgtcagcgatgctggcgaaagtctgg<br>Reverse: ccagactttcgccagcatcgctgacatacag
H478A	Forward: gaacggtgtctatctggctggtatgccaaatgc<br>Reverse: gcatttggcataccagccagatagacaccgttc
R184A/K187A	Forward: ccagtttctttgccgtgcatgcgccgctgc<br>Reverse: gcagcggcgcatgcacggcaaagaaactgg
K187A/R190A	Forward: gcgtgcatgcgccgctggctagctatgtc<br>Reverse: gacatagctagccagcggcgcatgcacgc
R190A/K204A	Forward: cgctggctagctatgtcaatctactcggtgggtgcacttgc<br>Reverse: gcaagtgcacccaccgagtagattgacatagctagccagcg

Amino acid sequence of His-tagged (N-terminal) MCR-1:

MHHHHHHHHHHSSGVDLGTENLYFQSNAGGGSGGGSMMQHTSVWYRRSVSPFVLVASVAVFLTATANLTF
FDKISQTYPIADNLGFVLTIAVVLFGAMLLITTLLSSYRYVLKPVLILLLIMGAVTSYFTDTYGTVYDTT
MLQNALQTDQAETKDLLNAAFIMRIIGLGVLPSLLVAFVKVDYPTWGKGLMRRLGLIVASLALILLPVVA
FSSHYASFFRVHKPLRSYVNPIMPIYSVGKLASIEYKKASAPKDTIYHAKDAVQATKPDMRKPRLVVFVV
GETARADHVSFNGYERDTFPQLAKIDGVTNFSNVTSCGTSTAYSVPCMFSYLGADEYDVDTAKYQENVLD
TLDRLGVSILWRDNNSDSKGVMDKLPKAQFADYKSATNNAICNTNPYNECRDVGMLVGLDDFVAANNGKD
MLIMLHQMGNHGPAYFKRYDEKFAKFTPVCEGNELAKCEHQSLINAYDNALLATDDFIAQSIQWLQTHSN
AYDVSMLYVSDHGESLGENGVYLHGMPNAFAPKEQRSVPAFFWTDKQTGITPMATDTVLTHDAITPTLLK
LFDVTADKVKDRTAFIR

Supplementary Table 3. List of additional primers.

Name	Sequence	Purpose
H10EptABHI<br>EptAERI	taatcgcGGATCCAAGAAGGAGATATACATGCATCATCACCATCACCATCACCACCATCACTTGAAGCGCCTACTAAAAAGACCCTC<br>atttaaccggaattccggtcaTTCACTCACTCTCCTGCAAGTTTGCAG	To create His10EptA. EptA template from W3110
F1<br>R1	CGGAGCGAATTCATATTCATATGGACCATGGCTAATTCC<br>TTATCCGGAATTCCGAAGTTCCTATTCTCTAGAAAG	EcoRI restriction site insertion in pKD4, to create pKDE
F2<br>R2	TTATATCCGGAATTCTAGAGCACAGCTAACACCAC<br>GAATTCCATATGGTACTTTCCTGTGTGACTC	Amplification of EcoRI-iP-RBS-NdeI, to create pKDE-iP
F3<br>R3	TGTGTAGGCTGGAGCTGCTTCG<br>CATATGGTACTTTCCTGTGTGACTC	Amplification of P1-FRT-Kan-FRT-iP-RBS (kan-lpxE)
F5<br>R5	ACCGCGTAAGGTTGCCTGCGTTTTTCAGTAAGATAATTAGAGAAAATATGGTGTAGGCTGGAGCTGCTTC<br>ATCCTCACTATAAAAATAACCCTGATGATGTTAATTACTGTGAGTTATTTCTAAATAATCTCTCTATTTCTC	Amplifies lpxE ORF including iP-RBS overlapping region
Up-ver-lpxE<br>Dw-ver-lpxE	ACTACCGCGTAAGGTTGCCT<br>TCCTCACTATAAAAATAACC	Verification of lpxT replacement by lpxE
F6<br>R6	GAGTCACACAGGAAAGTACCATATGGCAAGATTTCATATCATATTAGGTTTAGTTG<br>TCAATATTCTTTTTTACGATACATTAGTGCATAAACC	Amplifies lpxF ORF including IP-RB S overlapping region
F7<br>R7	ATCACCAGATTGATTTTTGCCTTATCCGAAACTGGAAAAGCATGGAAACGTGTGTAGGCTGGAGCTGCTTCG<br>CAGGCGAAGGCCTCTCCTCGCGAGAGGCTTTTTTATTTGATGGGATAAAGTCAATATTCTTTTTTACGATACATTAGTGCATAAACC	Amplification of [H1lpxM-P1-FRT- Kan-iP-RBS-LpxF-H2lpxM]
Up-ver-lpxF<br>Dw-ver-lpxF	GCAGGCCAAAGAGATTGTG<br>CCACGCGTATTTTAACGGTAG	Verification of lpxM replacement by LpxF
MCR-1 pMCSG7 F<br>MCR-1 pMCSG7 R	tacttccaatccaatgccATGATGCAGCATACTTCTGTGTG<br>ttatccacttccaatgtcaGCGGATGAATGCGGTGC	To generate N-terminally His-tagged MCR-1

Supplementary Table 4. Summary of strains and plasmids.

Strains	Description	Source
W3110	Wild type, F-I- rph-1 INV(rrnD, rrnE)1 rph-1	*E. coli* Genetic stock center Yale
DY330	W3110 ΔlacU169 gal490 λ cI857 Δ(cro-bioA)	Yu *et al.*[83]
BN0	W3110 ΔlpxT ΔeptA, camR, kanR	Needham *et al.*[14]
W3lpxT::LpxE+ ΔeptA	W3110 ΔlpxT::lpxEFn ΔeptA, kanR	This study
BN1	ΔlpxT ΔeptA ΔpagP	Needham *et al.*[14]
BN1 lpxM:: LpxF	BN1 lpxM::lpxF, KanR	This study

Plasmids		
pUC-iP	pUC57 containing a 159-bp proD insulated promoter sequence, AmpR	This study
pKD4	nptII gene template plasmid, KanR, AmpR	Datsenko *et al.*[84]
pCP20	FLP-expressing vector, AmpR, CamR	Datsenko *et al.*[84]
pKDE	Modified pKD4 with an EcoRI site at 1483, kanR, AmpR	This study
pKDE-iP	pKDE containing a proD insulated promoter-RBS between EcoR-NdeI, KanR, AmpR	This study
pQLpxE	pQLINK vector containing lpxE ORF from Francisella novicida, AmpR	Needham *et al.*[14]
pQLpxF	pQLINK vector containing lpxF ORF from Francisella novicida, AmpR	Needham *et al.*[14]
pWSK29	Low copy number plasmid, AmpR	Wang *et al.*[79]
pWHisEptAEc	pWSK29 containing His10-eptA at N-terminal	This study
pWMcr1	pWSK29 containing phosphoethanolamine transferase mcr1	This study
pWHisMcr1	pWSK29 containing His10-mcr-1 at N-terminal	This study
pACYC184	pA15A origin of replication, low copy number cloning vector, TetR, CamR	Novagen
pACeptAPa	pACYC184 containing phosphoethanolamine transferase eptA from P. aeruginosa	Nowicki *et al.*[51]
pMCSG7	High copy number plasmid, AmpR	Stols *et al.*[59]

Supplementary Table 5. Summary of the MD simulations.

System	KLA	POPE	Zn^{2+}	Modification	# Water	# POPE	# POPG
Apo_MCR-1 (State 1)	--	--	--	--	54364	424	106
Apo_MCR-1 (State 2)	--	--	--	--	69912	501	125
Apo_MCR-1_K401A (State 1)	--	--	--	K401A	54168	422	104
Apo_MCR-1_R402A (State 1)	--	--	--	R402A	54392	423	105
Apo_MCR-1_D119A (State 1)	--	--	--	D119A	54456	425	106
MCR-1_PE_KLA	yes	yes	--	--	54170	422	104
MCR-1_PE_KLA_ZN	yes	yes	yes	--	54168	422	104
PEtN-T285_MCR-1_KLA_ZN (State 1)	yes	--	yes	PEtN-T285	54166	422	104
PEtN-T285_MCR-1_KLA_ZN (State 2)	yes	--	yes	PEtN-T285	61298	469	115

Supplementary References

1 Miller, K. R. *et al.* T cell receptor-like recognition of tumor in vivo by synthetic antibody fragment. *PLoS One* **7**, e43746 (2012). https://doi.org:10.1371/journal.pone.0043746

2 Rizk, S. S. *et al.* Allosteric control of ligand-binding affinity using engineered conformation-specific effector proteins. *Nat Struct Mol Biol* **18**, 437-442 (2011). https://doi.org:10.1038/nsmb.2002

3 Fellouse, F. A. *et al.* High-throughput generation of synthetic antibodies from highly functional minimalist phage-displayed libraries. *J Mol Biol* **373**, 924-940 (2007). https://doi.org:10.1016/j.jmb.2007.08.005

4 Punjani, A., Rubinstein, J. L., Fleet, D. J. & Brubaker, M. A. cryoSPARC: algorithms for rapid unsupervised cryo-EM structure determination. *Nat Methods* **14**, 290-296 (2017). https://doi.org:10.1038/nmeth.4169

5 Rubinstein, J. L. & Brubaker, M. A. Alignment of cryo-EM movies of individual particles by optimization of image translations. *J Struct Biol* **192**, 188-195 (2015). https://doi.org:10.1016/j.jsb.2015.08.007

6 Anandan, A. *et al.* Structure of a lipid A phosphoethanolamine transferase suggests how conformational changes govern substrate binding. *Proc Natl Acad Sci U S A* **114**, 2218-2223 (2017). https://doi.org:10.1073/pnas.1612927114

7 Hinchliffe, P. *et al.* Insights into the Mechanistic Basis of Plasmid-Mediated Colistin Resistance from Crystal Structures of the Catalytic Domain of MCR-1. *Sci Rep* **7**, 39392 (2017). https://doi.org:10.1038/srep39392

8 Nowicki, E. M., O'Brien, J. P., Brodbelt, J. S. & Trent, M. S. Extracellular zinc induces phosphoethanolamine addition to Pseudomonas aeruginosa lipid A via the ColRS two-component system. *Mol Microbiol* **97**, 166-178 (2015). https://doi.org:10.1111/mmi.13018
