# Supplementary material for: Mechanistic basis of antimicrobial resistance mediated by the phosphoethanolamine transferase MCR-1
Source: Nat Commun. 2025 Nov 26;16:10516. doi: 10.1038/s41467-025-65515-3 (PMC12658134; doi:10.1038/s41467-025-65515-3)
Supplement: Supplementary file 2 — Description of Additional Supplementary Files [file 41467_2025_65515_MOESM2_ESM.pdf]

Description of Additional Supplementary Files:

Supplementary Movie 1: Movement of MCR-1 periplasmic domain in apo state.

Supplementary Movie 2: Stabilization of PE in the active site by Zn2+

Supplementary Movie 3: MCR-1 K401A mutant showing transition from State 1 to State 2.
