# Supplementary material for: Mechanistic basis of antimicrobial resistance mediated by the phosphoethanolamine transferase MCR-1
Source: Nat Commun. 2025 Nov 26;16:10516. doi: 10.1038/s41467-025-65515-3 (PMC12658134; doi:10.1038/s41467-025-65515-3)
Supplement: Supplementary file 6 — Reporting Summary [file 41467_2025_65515_MOESM6_ESM.pdf]

nature portfolio

Corresponding author(s): Filippo Mancia (fm123@cumc.columbia.edu), Phillip J. Stansfeld (Phillip.Stansfeld@warwick.ac.uk), Rie Nygaard (rin7007@med.cornell.edu), & M. Stephen Trent (strent@uga.edu)

Last updated by author(s): October 3, 2025

Reporting Summary

Nature Portfolio wishes to improve the reproducibility of the work that we publish. This form provides structure for consistency and transparency in reporting. For further information on Nature Portfolio policies, see our Editorial Policies and the Editorial Policy Checklist.

Statistics

For all statistical analyses, confirm that the following items are present in the figure legend, table legend, main text, or Methods section.

n/a	Confirmed	
x		The exact sample size (*n*) for each experimental group/condition, given as a discrete number and unit of measurement
	x	A statement on whether measurements were taken from distinct samples or whether the same sample was measured repeatedly
x		The statistical test(s) used AND whether they are one- or two-sided<br>*Only common tests should be described solely by name; describe more complex techniques in the Methods section.*
x		A description of all covariates tested
	x	A description of any assumptions or corrections, such as tests of normality and adjustment for multiple comparisons
x		A full description of the statistical parameters including central tendency (e.g. means) or other basic estimates (e.g. regression coefficient) AND variation (e.g. standard deviation) or associated estimates of uncertainty (e.g. confidence intervals)
x		For null hypothesis testing, the test statistic (e.g. *F*, *t*, *r*) with confidence intervals, effect sizes, degrees of freedom and *P* value noted<br>*Give P values as exact values whenever suitable.*
x		For Bayesian analysis, information on the choice of priors and Markov chain Monte Carlo settings
x		For hierarchical and complex designs, identification of the appropriate level for tests and full reporting of outcomes
x		Estimates of effect sizes (e.g. Cohen's *d*, Pearson's *r*), indicating how they were calculated

Our web collection on statistics for biologists contains articles on many of the points above.

Software and code

Policy information about availability of computer code

Data collection	Leginon 3.5 (for cryo-EM data collection)
Data analysis	cryoSPARC v.4.0.1 (for analyzing and processing cryo-EM data), Namdinator (for model fitting), Coot v0.9.8.7 (for manual fitting of the model into the map), PHENIX v1.21.2 (for fitting of ligands into the map), UCSF Chimera v1.19 (for visualizing the structures), UCSF ChimeraX v1.10 (for visualizing the structures), GROMACS 2022 (for MD simulations), MEMEMBED v1.15 (for MD simulations), Martini v3 (for MD simulations), CG2AT (for MD simulations), CHARMM36m (for MD simulations), GROMACS 2022 tools (for analyzing the MD simulation data), MDAnalysis v2.8.0 (for analyzing the MD simulation data), GraphPrism 7 (for ELISA data analysis)

For manuscripts utilizing custom algorithms or software that are central to the research but not yet described in published literature, software must be made available to editors and reviewers. We strongly encourage code deposition in a community repository (e.g. GitHub). See the Nature Portfolio guidelines for submitting code & software for further information.

Data

Policy information about availability of data

All manuscripts must include a data availability statement. This statement should provide the following information, where applicable:

- Accession codes, unique identifiers, or web links for publicly available datasets
- A description of any restrictions on data availability
- For clinical datasets or third party data, please ensure that the statement adheres to our policy

The Cryo-EM density map has been deposited into the Electron Microscopy Data Bank (EMDB) under accession code EMD-49896. The model has been deposited in the Protein Data Bank (PDB) under accession code 9NWW. The MD simulation data generated in this study are available as a supplementary dataset on Zenodo (https://doi.org/10.5281/zenodo.17258531). All raw gels and full TLC plate images are available in the Supplementary Information. Source data are provided with this paper.

Research involving human participants, their data, or biological material

Policy information about studies with human participants or human data. See also policy information about sex, gender (identity/presentation), and sexual orientation and race, ethnicity and racism.

Reporting on sex and gender	This information has not been collected.
Reporting on race, ethnicity, or other socially relevant groupings	This information has not been collected.
Population characteristics	This information has not been collected.
Recruitment	This information has not been collected.
Ethics oversight	This information has not been collected.

Note that full information on the approval of the study protocol must also be provided in the manuscript.

Field-specific reporting

Please select the one below that is the best fit for your research. If you are not sure, read the appropriate sections before making your selection.

☒ Life sciences ☐ Behavioural & social sciences ☐ Ecological, evolutionary & environmental sciences

For a reference copy of the document with all sections, see nature.com/documents/nr-reporting-summary-flat.pdf

Life sciences study design

All studies must disclose on these points even when the disclosure is negative.

Sample size	The single-point phage ELISA (Supplementary Fig. 2c) is the initial screening after phage display. Typically there are 96-192 "single points" - each representing individual E. coli colonies from which phage were amplified. The 96-192 "single points" is a representative set of these samples (Supplementary Fig. 2c). We then take all the "positive clones" - those giving antigen-specific ELISA signals and low-to-no background/nanodisc signals - and sequence them. It is at this point that we know which sequences are unique; often each clone is represented multiple times in this pool as the specific binders have been enriched through several rounds of biopanning. These unique clones are then sub-cloned into expression vectors, expressed, purified, and tested again for binding affinity, this time as purified Fabs.
Data exclusions	No data was excluded from the analyses.
Replication	For reproducibility, all SDS-PAGE gels were run in duplicate (Supplementary Fig. 1e, Supplementary Fig. 7a), and TLC data is representative of three biological replicates.
Randomization	Not relevant - no samples or organisms were allocated into groups for this study. All ELISA data analyzed single points and no data was excluded.
Blinding	Blinding was not relevant to this study, as no research coordinator was instated to oversee the blinding process of the different experimental approaches performed in the different participating labs of this collaborative study.

Behavioural & social sciences study design

All studies must disclose on these points even when the disclosure is negative.

Study description	N/A

Research sample	N/A
Sampling strategy	N/A
Data collection	N/A
Timing	N/A
Data exclusions	N/A
Non-participation	N/A
Randomization	N/A

Ecological, evolutionary & environmental sciences study design

All studies must disclose on these points even when the disclosure is negative.

Study description	N/A
Research sample	N/A
Sampling strategy	N/A
Data collection	N/A
Timing and spatial scale	N/A
Data exclusions	N/A
Reproducibility	N/A
Randomization	N/A
Blinding	N/A

Did the study involve field work? ☐ Yes ☒ No

Field work, collection and transport

Field conditions	N/A
Location	N/A
Access & import/export	N/A
Disturbance	N/A

Reporting for specific materials, systems and methods

We require information from authors about some types of materials, experimental systems and methods used in many studies. Here, indicate whether each material, system or method listed is relevant to your study. If you are not sure if a list item applies to your research, read the appropriate section before selecting a response.

Materials & experimental systems

n/a	Involved in the study	
☐	☒	Antibodies
☒	☐	Eukaryotic cell lines
☒	☐	Palaeontology and archaeology
☒	☐	Animals and other organisms
☒	☐	Clinical data
☒	☐	Dual use research of concern
☒	☐	Plants

Methods

n/a	Involved in the study	
☒	☐	ChIP-seq
☒	☐	Flow cytometry
☒	☐	MRI-based neuroimaging

Antibodies

Antibodies used

Horseradish peroxidase HRP-conjugated anti M13-monoclonal antibody (GE Healthcare #27-9421-01: diluted 1:5,000)
HRP conjugated mouse anti-human IgG F(ab')2 monoclonal antibody (Jackson cat# AB_2339089; diluted 1:5,000)

Validation

Horseradish peroxidase HRP-conjugated anti M13-monoclonal antibody (GE Healthcare) has been validated by the manufacturer for ELISA use. Further information is available at https://www.sigmaaldrich.com/US/en/product/sigma/ge27942101?gclid=Cj0KCQiA_8OPBhDtARIsAKQu0gZVs1ytwIFRY4MuPvKBucg4idFxfUZ6XIBEDAoMLTxYRNaYRkS-RpcaApqAEALw_wcB

HRP conjugated mouse anti-human IgG F(ab')2 monoclonal antibody (Jackson cat# AB_2339089) has been validated for western blot with 7 publications by the manufacturer. More information is available at htpps://jacksonimmuno.com/catalog/products/109-035-006

Eukaryotic cell lines

Policy information about cell lines and Sex and Gender in Research

Cell line source(s): N/A

Authentication: N/A

Mycoplasma contamination: N/A

Commonly misidentified lines (See ICLAC register): N/A

Palaeontology and Archaeology

Specimen provenance: N/A

Specimen deposition: N/A

Dating methods: N/A

☐ Tick this box to confirm that the raw and calibrated dates are available in the paper or in Supplementary Information.

Ethics oversight: N/A

Note that full information on the approval of the study protocol must also be provided in the manuscript.

Animals and other research organisms

Policy information about studies involving animals; ARRIVE guidelines recommended for reporting animal research, and Sex and Gender in Research

Laboratory animals: The study did not involve the use of laboratory animals.

Wild animals: The study did not involve the use of wild animals.

Reporting on sex: N/A

Field-collected samples: N/A

Ethics oversight: N/A

Note that full information on the approval of the study protocol must also be provided in the manuscript.

Clinical data

Policy information about clinical studies

All manuscripts should comply with the ICMJE guidelines for publication of clinical research and a completed CONSORT checklist must be included with all submissions.

Clinical trial registration	N/A
Study protocol	N/A
Data collection	N/A
Outcomes	N/A

Dual use research of concern

Policy information about dual use research of concern

Hazards

Could the accidental, deliberate or reckless misuse of agents or technologies generated in the work, or the application of information presented in the manuscript, pose a threat to:

No	Yes	
☒	☐	Public health
☒	☐	National security
☒	☐	Crops and/or livestock
☒	☐	Ecosystems
☒	☐	Any other significant area

Experiments of concern

Does the work involve any of these experiments of concern:

No	Yes	
☒	☐	Demonstrate how to render a vaccine ineffective
☒	☐	Confer resistance to therapeutically useful antibiotics or antiviral agents
☒	☐	Enhance the virulence of a pathogen or render a nonpathogen virulent
☒	☐	Increase transmissibility of a pathogen
☒	☐	Alter the host range of a pathogen
☒	☐	Enable evasion of diagnostic/detection modalities
☒	☐	Enable the weaponization of a biological agent or toxin
☒	☐	Any other potentially harmful combination of experiments and agents

Plants

Seed stocks	N/A
Novel plant genotypes	N/A
Authentication	N/A

ChIP-seq

Data deposition

☐ Confirm that both raw and final processed data have been deposited in a public database such as GEO.

☐ Confirm that you have deposited or provided access to graph files (e.g. BED files) for the called peaks.

Data access links<br>*May remain private before publication.*	N/A
Files in database submission	N/A
Genome browser session<br>(e.g. UCSC)	N/A

Methodology

Replicates	N/A
Sequencing depth	N/A
Antibodies	N/A
Peak calling parameters	N/A
Data quality	N/A
Software	N/A

Flow Cytometry

Plots

Confirm that:

☐ The axis labels state the marker and fluorochrome used (e.g. CD4-FITC).

☐ The axis scales are clearly visible. Include numbers along axes only for bottom left plot of group (a 'group' is an analysis of identical markers).

☐ All plots are contour plots with outliers or pseudocolor plots.

☐ A numerical value for number of cells or percentage (with statistics) is provided.

Methodology

Sample preparation	N/A
Instrument	N/A
Software	N/A
Cell population abundance	N/A
Gating strategy	N/A

☐ Tick this box to confirm that a figure exemplifying the gating strategy is provided in the Supplementary Information.

Magnetic resonance imaging

Experimental design

Design type	N/A
Design specifications	N/A
Behavioral performance measures	N/A

Acquisition

Imaging type(s)	N/A
Field strength	N/A
Sequence & imaging parameters	N/A
Area of acquisition	N/A

Diffusion MRI ☐ Used ☒ Not used

Preprocessing

Preprocessing software	N/A
Normalization	N/A
Normalization template	N/A
Noise and artifact removal	N/A
Volume censoring	N/A

Statistical modeling & inference

Model type and settings	N/A
Effect(s) tested	N/A

Specify type of analysis: ☐ Whole brain ☐ ROI-based ☐ Both

Statistic type for inference (See Eklund et al. 2016)	N/A
Correction	N/A

Models & analysis

n/a	Involved in the study
☒	☐ Functional and/or effective connectivity
☒	☐ Graph analysis
☒	☐ Multivariate modeling or predictive analysis

Functional and/or effective connectivity	N/A
Graph analysis	N/A
Multivariate modeling and predictive analysis	N/A
