# Supplementary material for: Mechanistic basis of antimicrobial resistance mediated by the phosphoethanolamine transferase MCR-1
Source: Nat Commun. 2025 Nov 26;16:10516. doi: 10.1038/s41467-025-65515-3 (PMC12658134; doi:10.1038/s41467-025-65515-3)
Supplement: Supplementary file 8 — Source Data [file 41467_2025_65515_MOESM8_ESM.zip › SourceData/SourceData.docx]

**Source data:**

***Red, dashed boxes indicate how gels were cropped.**

**Figure 2b | Uncropped TLC plates from Main Figure.** TLC analysis from Fig. 2b of lipid A isolated from cells expressing MCR-1 mutants near the PE binding site, on left, and KLA binding site, on right.

**Supplementary Figure 1b | Uncropped TLC plate from Supplementary Figure 1b.** TLC analysis from Supplementary Fig. 1b of lipid A isolated from cells expressing MCR-1 WT (N-terminal His-tag).

**Supplementary Figure 1d | Nanodisc incorporation experiment.** HPLC elution curves for MCR-1 solubilized in DDM detergent (blue), and reconstituted in the following nanodiscs: MSP1D1 + POPC (orange), MSP1D1 + POPG (green), MSP1D1 + *E. coli* Polar Extract lipids (light blue), MSP1E3D1 + POPC (purple), MSP1E3D1 + POPG (light green), and MSP1E3D1 + *E. coli* Polar Extract lipids (dark blue).

**Supplementary Figure 1e | Uncropped SDS-PAGE gel from Supplementary Figure 1e.** SDS-PAGE gel from Supplementary Fig. 1e depicting MCR-1 purified in detergent, MCR-1 reconstituted into nanodiscs (MSP1D1 and POPG), and MCR-1 reconstituted into nanodiscs (MSP1D1 and POPG) with Fab (MR6) bound. Molecular weight markers can also be observed and are labeled.

**Supplementary Figure 7a | Uncropped SDS-PAGE gels from Supplementary Figure 7a.** SDS-PAGE gels from Supplementary Fig. 7a of all MCR-1 mutants used for TLC functional analysis, which were purified to verify expression.

**Supplementary Figure 11a | Uncropped TLC plate from Supplementary Figure 11a.** TLC analysis from Supplementary Fig. 11a of bis-phosphorylated, 1-dephosphorylated, and 4'-dephosphorylated lipid A isolated from cells expressing MCR-1, EptA from *E. coli* or *P. aeruginosa*, or empty vector (pWSK29).

**
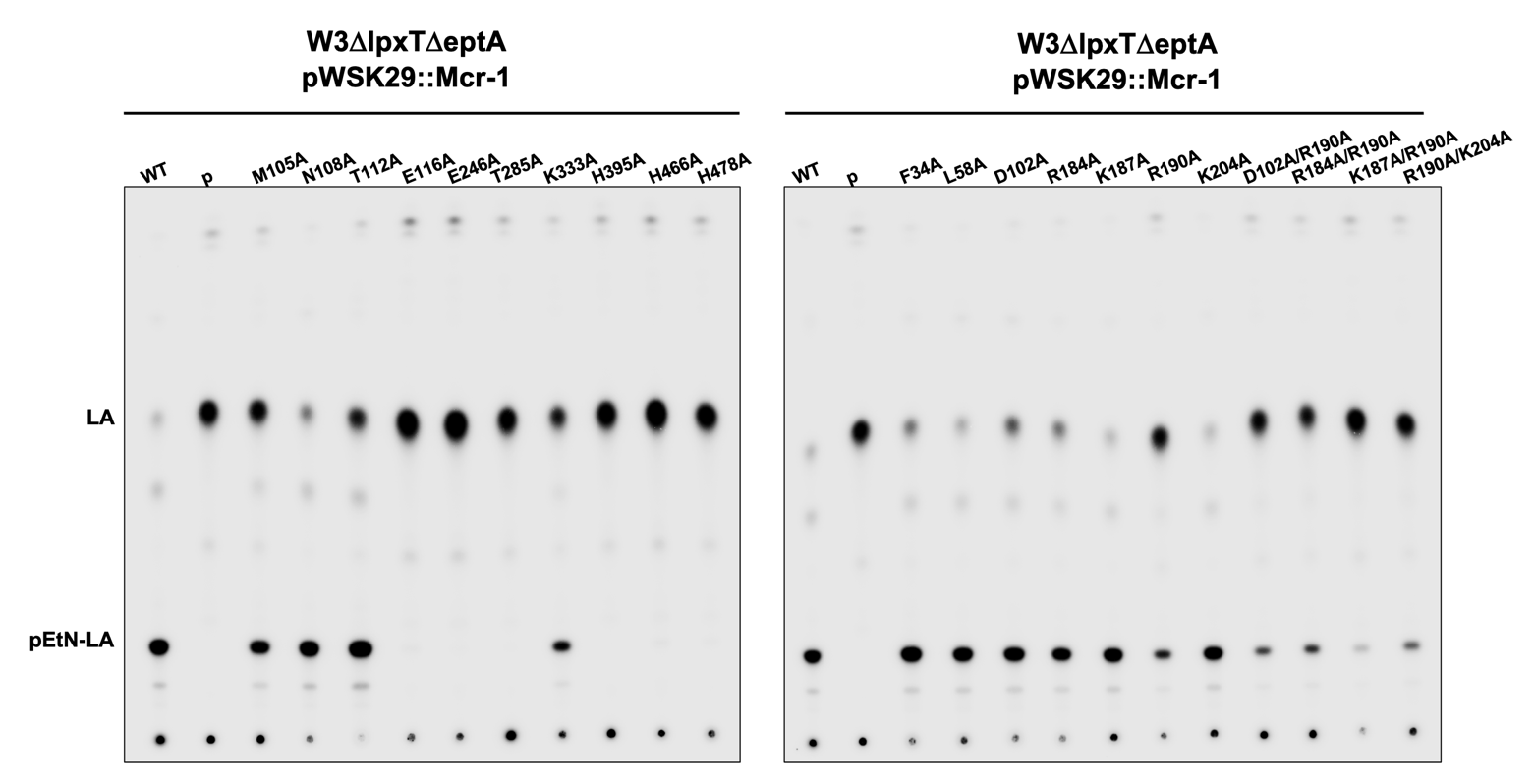
**

**Figure 2b | Uncropped TLC plates from Main Figure.**

**
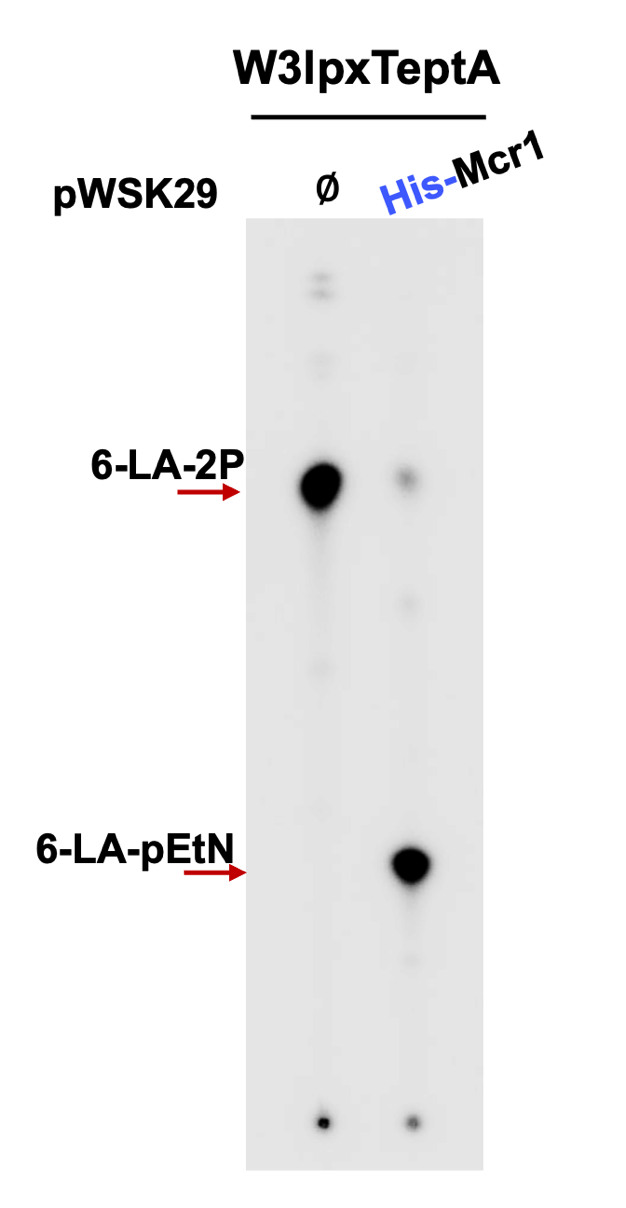
**

**Supplementary Figure 1b | Uncropped TLC plate from Supplementary Figure 1b.**

**
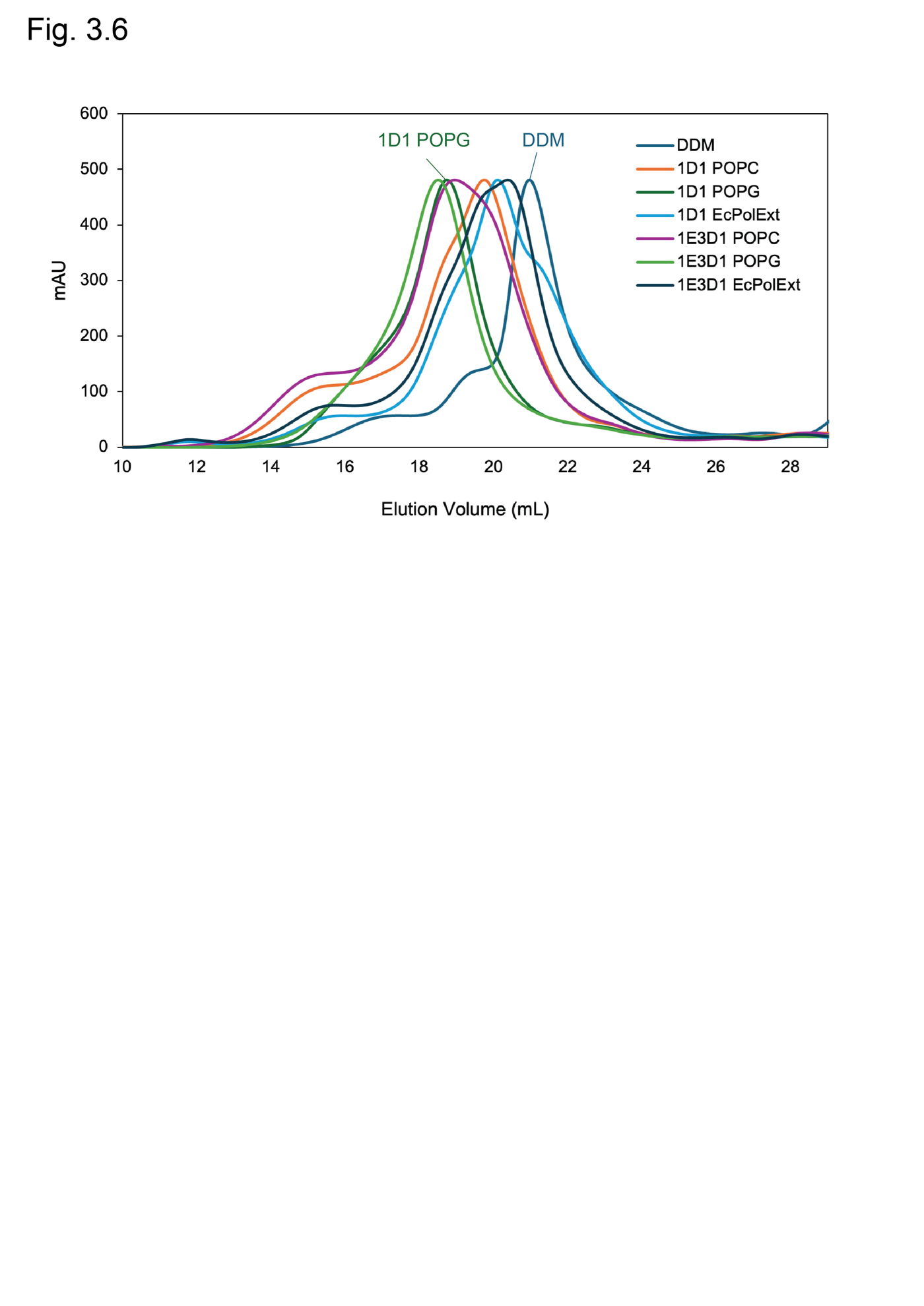
 Supplementary Figure 1d | Nanodisc incorporation experiment.**

**
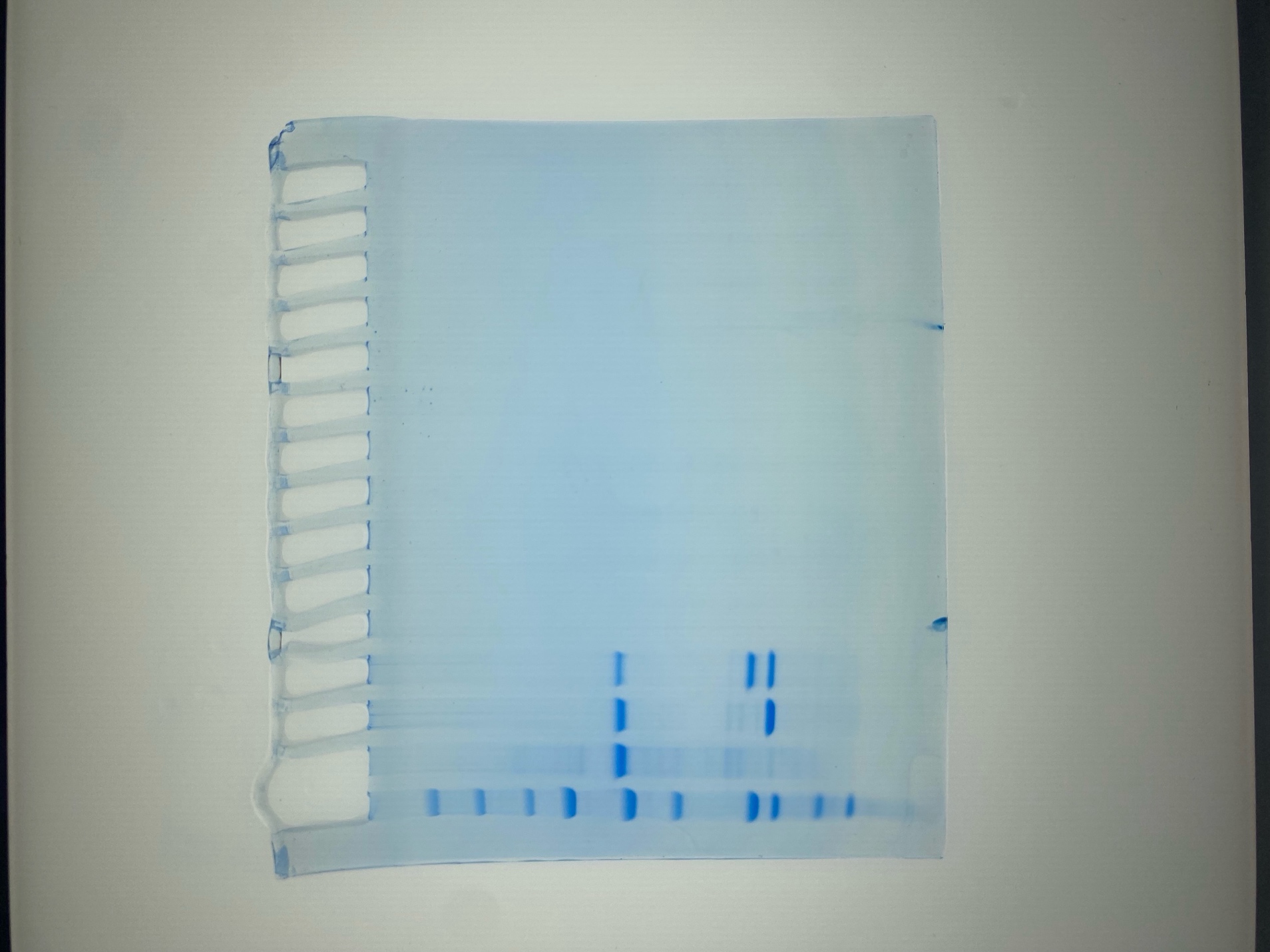
**

**Supplementary Figure 1e | Uncropped SDS-PAGE gel from Supplementary Figure 1e.**


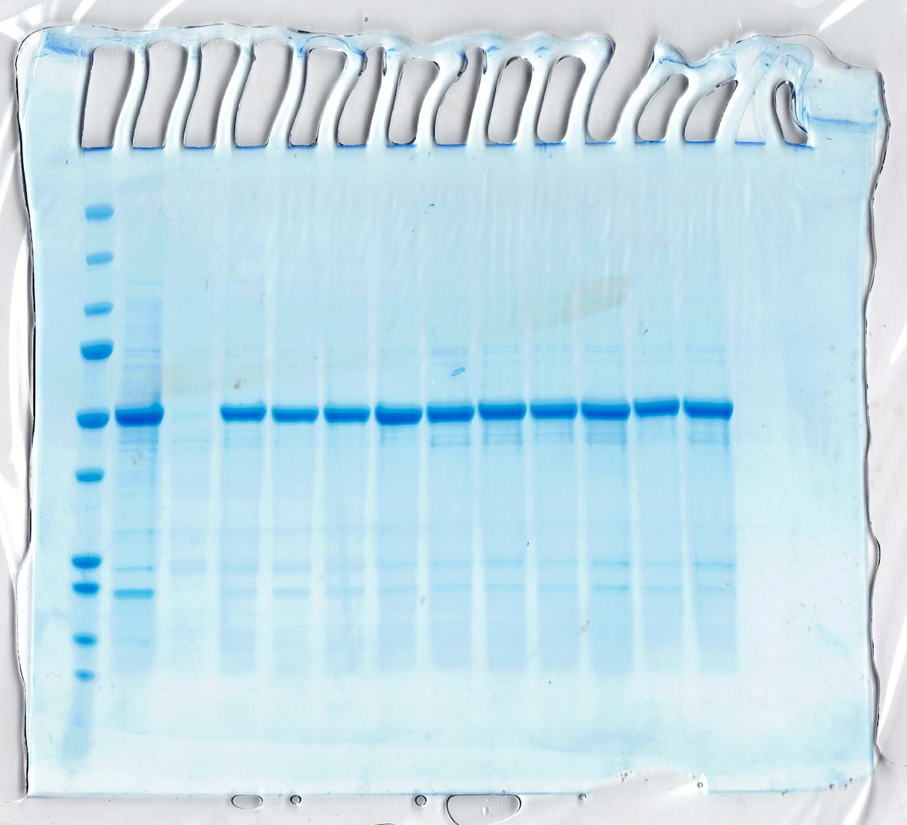


**
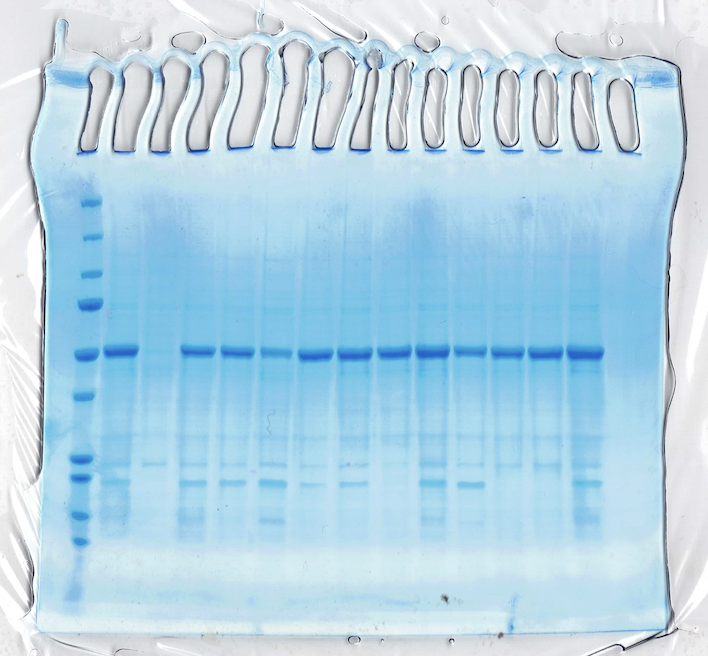
**

**Supplementary Figure 7a | Uncropped SDS-PAGE gels from Supplementary Figure 7a.**

**
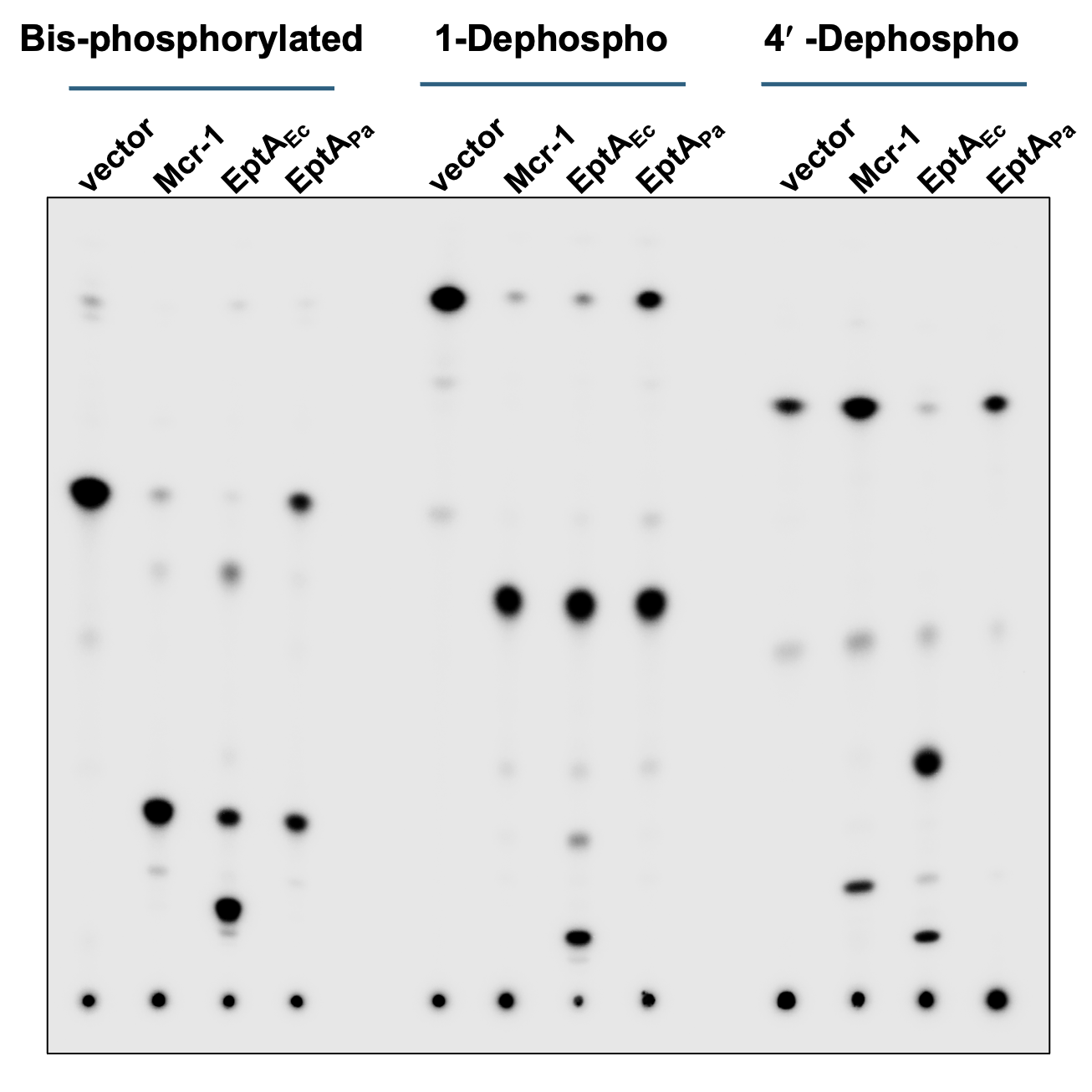
**

**Supplementary Figure 11a | Uncropped TLC plate from Supplementary Figure 11a.**
